# Molecular regionalization in the compact brain of the meiofaunal annelid *Dinophilus gyrociliatus* (Dinophilidae)

**DOI:** 10.1186/s13227-016-0058-2

**Published:** 2016-08-30

**Authors:** Alexandra Kerbl, José M. Martín-Durán, Katrine Worsaae, Andreas Hejnol

**Affiliations:** 1 Marine Biology Section, Department of Biology, Faculty of Science, University of Copenhagen, Universitetsparken 4, 2100 Copenhagen, Denmark; 2 Sars International Centre for Marine Molecular Biology, University of Bergen, Thormøhlensgate, 55, 5006 Bergen, Norway

**Keywords:** Meiofauna, Direct development, Brain, Nervous system, Annelida, Larva, Animal evolution

## Abstract

**Background:**

Annelida is a morphologically diverse animal group that exhibits a remarkable variety in nervous system architecture (e.g., number and location of longitudinal cords, architecture of the brain). Despite this heterogeneity of neural arrangements, the molecular profiles related to central nervous system patterning seem to be conserved even between distantly related annelids. In particular, comparative molecular studies on brain and anterior neural region patterning genes have focused so far mainly on indirect-developing macrofaunal taxa. Therefore, analyses on microscopic, direct-developing annelids are important to attain a general picture of the evolutionary events underlying the vast diversity of annelid neuroanatomy.

**Results:**

We have analyzed the expression domains of 11 evolutionarily conserved genes involved in brain and anterior neural patterning in adult females of the direct-developing meiofaunal annelid *Dinophilus gyrociliatus*. The small, compact brain shows expression of *dimmed*, *foxg*, *goosecoid*, *homeobrain*, *nk2.1*, *orthodenticle*, *orthopedia*, *pax6*, *six3*/*6* and *synaptotagmin*-*1*. Although most of the studied markers localize to specific brain areas, the genes *six3*/*6* and *synaptotagmin*-*1* are expressed in nearly all perikarya of the brain. All genes except for *goosecoid*, *pax6* and *nk2.2* overlap in the anterior brain region, while the respective expression domains are more separated in the posterior brain.

**Conclusions:**

Our findings reveal that the expression patterns of the genes *foxg*, *orthodenticle*, *orthopedia* and *six3*/*6* correlate with those described in *Platynereis dumerilii* larvae, and *homeobrain*, *nk2.1*, *orthodenticle* and *synaptotagmin*-*1* resemble the pattern of late larvae of *Capitella teleta*. Although data on other annelids are limited, molecular similarities between adult *Dinophilus* and larval *Platynereis* and *Capitella* suggest an overall conservation of molecular mechanisms patterning the anterior neural regions, independent from developmental and ecological strategies, or of the size and configuration of the nervous system.

**Electronic supplementary material:**

The online version of this article (doi:10.1186/s13227-016-0058-2) contains supplementary material, which is available to authorized users.

## Background

Several detailed studies on the nervous systems of various groups within Annelida (“segmented worms”) demonstrate that this organ system displays a remarkable variability in arrangement and structure of the brain and neuropil, number of ventral nerve cords and nerves, as well as layout of the stomatogastric nervous system and peripheral nerves (e.g., [1,8]). The position and configuration of the brain vary among annelids, most commonly consisting of a subepidermal dorsal neuropil with peripheral perikarya, subdivided into discrete clusters or lobes [1, 5, 6, 9, 10], but occasionally situated intraepidermally (e.g., [11, 12]) and ventrally (e.g., [13–15]), or anteriorly [13], or having a uniform compact morphology without apparent compartmentalization [11, 12, 16–26]. Despite this morphological diversity, the underlying molecular patterns of neural-related genes that are evolutionarily conserved in Protostomia (e.g., Mollusca, Platyhelminthes, Nemertea, Nematoda, Arthropoda and Brachiopoda [16–26]) and Deuterostomia (e.g., Hemichordata and Chordata, e.g., [9, 27–30]) have been analyzed only in a handful of annelid taxa [23, 31–35]. For instance, the transcription factor *six3*/*6* and the gene *synaptotagmin*-*1* (coding for the eponymous membrane-trafficking protein) pattern the presumptive neuroectoderm and the larval brain in *Platynereis dumerilii* and *Capitella teleta* [23, 33, 34], while domains of the genes *foxg* and *orthodenticle* appear in close proximity to the locomotory cilia of the prototroch (see Table 1 for comparison [21–23, 36]). However, most of the annelid species investigated with regard to molecular patterning of the anterior neural region so far (e.g., *C. teleta* and *P. dumerilii*) are characterized by exhibiting an indirect development comprising a planktonic microscopic larva and a macroscopic adult [16, 22, 23, 37–40]. Information about these patterning mechanisms in Oligochaeta and Hirudinea is still limited (e.g., [35, 41–44], Table 1 and literature therein) and completely absent in direct-developing, microscopic interstitial groups such as Dinophilidae [45–48]. Neuroanatomical studies of developing and adult brains in these microscopic species are also very limited (e.g., [13, 45, 46, 49–51]). Therefore, investigations in members of annelid lineages with alternative life cycles, ecological strategies and neuroanatomies are essential to attain a better understanding of the diversification of the nervous system in Annelida (and Spiralia) and its underlying molecular patterning [52, 53].

**Table 1:** Expression patterns and assumed function of the genes investigated in this study in annelid representatives from reported literature [9, 15, 16, 18, 23, 33–36, 40–42, 44, 70, 74, 75, 84]

Gene	Species	Expression pattern	Proposed function	References
Goosecoid (*gsc*)	*Platynereis dumerilii* (trochophore larva)	Around the stomodeum, in the anterior foregut, parts of the stomodeal nervous system	Patterns roof of the foregut, marker for the anterior foregut and oral ectoderm	[75]
	*Capitella teleta* or sp. 1 (late larval stages)	Multiple cells at circumesophageal connectives (extend from each side of the brain in ventral–posterior direction laterally toward ventral nerve cord), bilateral pair in anterior ectoderm (several cells with distinctive elongate morphology)	Marks subset of anterior neurons associated with stomodeum and foregut, maybe involved in development and differentiation of circumesophageal connectives and neuronal subtype identity	[74]
Homeobrain (*hbn*)	*Capitella teleta* or sp. 1 (late larval stages)	Cells associated with epithelio–optic nerves and more medial cell clusters in the brain, in lateral epithelium clearly connected to eyes, between stages 6 and 8 medial brain cells expressing *hbnl* move closer together toward midline, later, two small clusters appear in anterior-most segment lateral to mouth. Expression levels appear to lower/in fewer cells/clusters in late stages and not present in juvenile stages	Differentiates larval eye and brain, confers identity of neuronal subsets, more restricted to brain in *Capitella* than in *Drosophila* (there also in the VNC)	[16]
Orthopedia (*otp*)	*Platynereis dumerilii* (trochophore larva)	Limited number of cells in the developing medial forebrain region comprising vasotocinergic neurons adjacent to large photoreceptor cilia and the RFamidergic neurons; peripherally, expression demarcates prototroch; also expressed in nerve cells in the apical organ	Demarcates the neuropeptidergic sensory-neurosecretory cells in apical organ	[18, 23]
Orthodenticle (*otx*)	*Platynereis dumerilii* (trochophore larva)	Most prominent in the oral region (stomodeum), along pre- and postoral ciliary bands, but also in cells of apical organ and few cells in the apical hemisphere, defining head region	Patterning pre- and postoral ciliary bands/loops	[75]
	*Capitella teleta* or sp. 1 (late larval stages)	Expressed in bilateral lobes of the brain, developing foregut, within the posterior growth zone of the segmented trunk (lateral–posterior ectoderm), within a few cells of the ventral nerve cord along midline; later (stage 9) expressed in the brain, foregut, ventral nerve cord and posterior growth zone	Involved in patterning/specifying oral ectoderm, endoderm, foregut and central nervous system in larval stages	[74]
	*Helobdella* sp.	At least three paralogs identified, limited to unsegmented head domain, expressed in central nervous system and foregut/surrounding the mouth opening at larval stage 8/9 and scattered cells in the epidermis, but not in the posterior trunk region		[41, 84]
	*Pristina leidyi* (regeneration)	Two paralogs (similar expression region, stronger and/or broader in otx2), otx2 in single medial cell of the ventral ganglia of fully formed midbody segments, extensively expressed during anterior regeneration, in late stages of regeneration adjacent to cerebral ganglia and foregut/pharynx, during fission also detected in the VNC	Definition of anterior structures during embryogenesis, involved in head development during regeneration and fission, involved in early processes of postembryonic head specification, possibly related to light-sensing organs	[42]
	*Hydroides elegans*	Early expression in the animal hemisphere, associated with ciliary bands in pre-metamorphic larvae (not in primary trochoblasts, but adjacent cells), also along midline (maybe as posterior sensory organ precursors)	Specification of neuronal populations	[36]
Paired box homeobox gene 6 (*pax6*)	*Platynereis dumerilii* (trochophore larva)	Bilateral patches of cells laterally around the prototroch in developing hemisphere and in ventral episphere; latter domains extend more dorsal than larval eyes—cells of the optic commissure, later also at the base of the differentiating eye; along the developing central nervous system of the body segments; neuroectoderm in trochophore larva	Patterns ectopic eyes and ventral neurogenic domain	[35, 40]
	*Helobdella* sp.	Two paralogs, pax6A: N teloblast lineage and few cells of O teloblast lineage generating majority of segmentally arranged, ganglionic neurons, in the head closely related to developing supraesophageal ganglion and surrounding tissues; eyes at dorsal lip of rostral sucker; head signal maintained throughout development, segmental expression fades at later stages; dorsally three longitudinal rows of segmentally repeated cells symmetrical lateral to dorsal midline	Widely conserved role in eye development, implication in CNS development	[44]
Forkhead box gene G (*foxg*)—brain factor 1 (*bf1*)	*Platynereis dumerilii* (trochophore larva)	Horseshoe-shaped domain in the brain, more lateral part represents the eye anlage during early development, in the brain expression retained during larval development	Coordinates activity of two opposing signaling centers patterning the telencephalon anlage: downstream of the ventral signal, Hh, to induce ventral (subpallial) identities and inhibits dorsal Wnt/b-catenin signaling through direct transcriptional repression of *Wnt8* which induces dorsal (pallial) identities in vertebrates	[9]
Six class gene 3/6 (*six3*/*6*)	*Platynereis dumerilii* (trochophore larva)	Almost the entire episphere, includes anlagen of antennae and palpae, surrounded by ring-like peristomial expression of *Pdu*-*otx* (covering equatorial/peristomial larval regions and overlapping with *six3* in the periphery of the episphere)	Covers the medial brain anlagen, includes a large part of the early differentiating neurosecretory cells	[34]
NK homeobox gene 2.1 (*nk2.1*)	*Platynereis dumerilii* (trochophore larva)	Medioanterior expression of *Pdu*-*nk2.1* laterally demarcated by *Pdu*-*pax6*	Subdividing the anterior body regions mediolaterally	[9, 18, 34]
	*Capitella teleta* or sp. 1 (late larval stages)	Two paralogs in stage 6/7: *nk2.1a*—subset of brain cells, dorsal–anterior foregut tissue, within the midgut and in rectum at posterior terminus of midgut; *nk2.1b*—two brain lobes, in subsurface cells of presumptive foregut, broad ventrolateral domain in trunk ectoderm and mesoderm, extending from posterior of the mouth to the telotroch	Involved in endoderm patterning and differentiation; patterns anterior ectoderm	[74]
NK homeobox gene 2.2 (*nk2.2*)	*Platynereis dumerilii* (trochophore larva)	Y-shaped pattern comprising slender domain of midline cells, demarcating medial edges of fusing neuroectoderm, strictly complementary to *pax6*	Patterns endoderm, together with other genes differentiating central nervous system	[35]
Neurosecretory differentiation factor dimmed (*dim*)	*Platynereis dumerilii* (trochophore larva)	Colocalized broadly with *otp* in apical organ neurons	Neuroendocrine transcription factor; differentiates neuroendocrine neurons; coexpressed with MIP in the median brain	[70]
Membrane-trafficking protein synaptotagmin-1 (*syt*)	*Platynereis dumerilii* (trochophore larva)	Demarcating specific differentiating neurons; restricted to basal cells; neuroectoderm comprises a progenitor zone containing postmitotic, nondifferentiated neuronal precursors and differentiation zone	Neuronal differentiation	[35]
	*Capitella teleta* or sp. 1 (late larval stages)	Most terminally differentiated neurons; expression pattern progresses from anterior to posterior and begins in the central nervous system; few cells on either side of the mouth; most, but not all of the forming ganglia in the ventral nerve cord; small number of cells along the dorsal midline; stomatogastric ganglia; single and small clusters of cells in the epidermis	Exocytosis of synaptic vesicles, neuronal differentiation	[33]
	*Lamellibrachia satsuma*	Brain and in the ventral nerve cord, laterally and dorsally encapsulated neuropils of the brain, neural cell bodies located lateral to giant axon, distinct tubular structure in vestimentiferan nervous system	Patterns nervous system	[15]

The majority of the genes tested in this study are located in the brain and/or the apical organ of adults and larval stages of the listed Annelida. The entire series of genes used in this study has not been assessed in any other annelid species

**Fig. 1:**
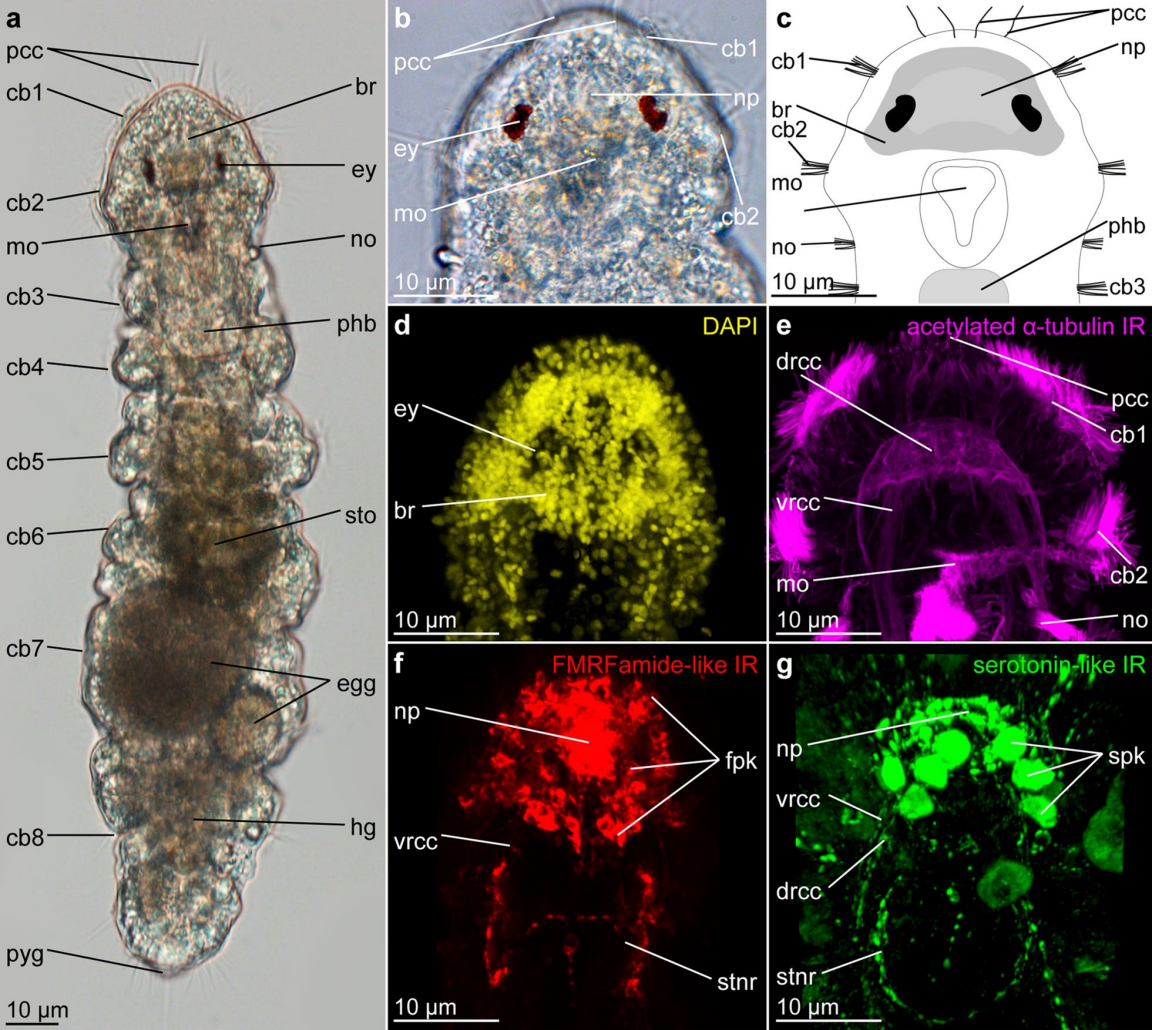
Morphology of the nervous system of adult female *Dinophilus gyrociliatus*. Light microscopic images of the adult female (**a**) and a detail of the head (**b**), which was subsequently used to create the template to map the gene expression patterns on **c**. **d**–**g** Details of the brain visualized by immunohistochemical labeling and confocal laser scanning microscopy of the direct labeling of DNA in the nuclei with DAPI (**d**), and the indirect labeling of acetylated α-tubulin-like immunoreactivity (**e**), FMRFamide-like immunoreactivity (**f**) and serotonin-like immunoreactivity (**g**). *br* brain, *cb1*–*8* ciliary bands 1–8, *drcc* dorsal root of the circumesophageal connective, *egg* eggs, *ey* eye, *fpk* perikarya with FMRFamide-like immunoreactivity, *mo* mouth opening, *no* nuchal organ, *np* neuropil, *pcc* prostomial compound cilia, *phb* pharyngeal bulb, *pyg* pygidium, *spk* perikarya with serotonin-like immunoreactivity, *stnr* stomatogastric nerve ring, *sto* stomach, *vrcc* ventral root of the circumesophageal connective

Dinophilidae is a species-poor meiofaunal group, whose members occupy interstitial habitats and lives in the crevices of sandy sediment or in biofilms on macroalgae in tidal marine regions [54–56]. It comprises two clades, *Dinophilus* and *Trilobodrilus*, whose representatives share their diminutive body size and being only few segments long pseudocoelomates with protonephridia, lacking chaetae, parapodia and appendages, but having a dense ventral ciliary field and direct development [45, 57–59]. Within *Dinophilus*, two morphotypes can be distinguished: one strongly pigmented, monomorphic group with prolonged life cycle and encystment period [55, 60] and a smaller, transparent, strongly dimorphic group with rapid life cycle [58, 61, 62]. The latter is represented by *D. gyrociliatus*, whose females (Fig. 1a) resemble the adults of the other morphotype to a certain degree, while the males are extremely miniaturized and short lived [61, 63, 64]. The brain of *Dinophilus gyrociliatus* consists of a compact neuropil formed by a dense meshwork of nerve fibers (Fig. 1b, c, e–g) with condensed fiber bundles probably representing commissures surrounded by a dense layer of perikarya (Fig. 1d, [45, 46]). The circumesophageal connective, which links the dorsal brain to the ventral nerve cords, bifurcates anteriorly at approximately the middle of the mouth opening into a ventral and a dorsal component (Fig. 1e). The ventral branch is connected to the stomatogastric nerve ring (a nervous loop dorsally lining the esophagus) and also gives rise to several nerve fibers innervating anterior compound cilia and at least the ventral pair of compound cilia at the anterior tip of the prostomium [2, 45, 46]. The dorsal branch gives rise to the paired dorsal, dorsolateral and lateral peripheral longitudinal nerves and the nerves innervating the nuchal organs [45, 46]. Approximately 750 densely packed, uniform perikarya surround the neuropil on the anterior, lateral, dorsal and posterior sides and very weak on the ventral side (Fig. 1d). The entire brain is located closely adjacent to the dorsal epidermis [45]. The eyes are sunken into the layer of brain perikarya (Fig. 1d) and seemingly directly connected to the neuropil without the formation of optic tracts [45, 58]. Since its nervous system and development are morphologically mapped in high detail [45, 46, 50, 58, 63, 64], *D. gyrociliatus* emerges as a suitable meiofaunal candidate next to the already established annelid models *P. dumerilii* and *C. teleta*.

In this study, we characterize the expression pattern of 11 genes with a putatively evolutionarily conserved role in patterning anterior neural regions in the brain of adult females of the microscopic *D. gyrociliatus*. These genes are generally associated with the anterior neurogenic domain (e.g., *six3*/*6*, *orthopedia*, *synaptotagmin*-*1*), ciliary bands (*foxg*) and sensory organs (*pax6*), or play a putative role in neurosecretory cell differentiation (*dimmed*) in previously investigated annelid and other invertebrate species (Table 1 and literature therein). We thus aim to investigate the possibility of a common molecular regionalization of the annelid brain, despite interspecific differences in the number of cells and morphological complexity, and provide a protocol for in situ hybridization approaches for adults in this microscopic annelid species.

## Methods

### Specimen rearing and fixation

Adult specimens of *Dinophilus gyrociliatus* Schmidt, 1848, are kept in culture in plastic boxes with seawater (28 per mille salinity) at 18 °C in the dark; water was exchanged twice per month. Spinach and fish food (Tetramin flakes for aquarium fish) were added to the cultures every 14 days (protocol modified from [65]). Mature animals were separated from the main cultures in small petri dishes and starved for 24–96 h. The specimens were fixed with 4 % paraformaldehyde in phosphate-buffered saline (PBS, pH 7.4) for one hour at room temperature directly after anesthetization with isotonic MgCl_2_. The fixative was removed by several rinses in 0.1 % PTw (PBS + 0.1 % Tween-20) before storing the specimens in 100 % methanol at −20 °C for later use.

### Gene cloning and orthology assignment

Putative orthologs of the genes of interest (*dimmed*, *foxg*, *goosecoid*, *homeobrain*, *nk2.1*, *nk2.2*, *orthodenticle*, *orthopedia*, *pax6*, *six3*/*6*, *synaptotagmin*-*1*) were identified by BLAST searches in the transcriptome of *D. gyrociliatus* using known sequences from other species as query. *D. gyrociliatus* transcriptome was constructed from approximately 300 specimens of mixed sexes and life stages, sequenced with Illumina technology and assembled with Trinity v.r20140717 using default settings [66]. The raw reads have been deposited at SRA: SRA Experiment: SRX2030658. The orthology of the genes was assigned by Bayesian phylogenetic analyses: amino acid sequences of *D. gyrociliatus* genes (GenBank Accession Numbers KX555473-KX555483) and orthologous proteins from other animals (Additional file 1, [22]) were aligned with MUSCLE [67], and the Bayesian phylogenetic analyses were performed on each dataset using MrBayes 3.2.6 [68], with settings described in [22]. Each analysis was run for 30,000,000 generations in four runs. A consensus tree and posterior probabilities for each branch were calculated and visualized in FigTree (http://tree.bio.ed.ac.uk/software/figtree/). Tracer (http://tree.bio.ed.ac.uk/software/tracer/) was used to test whether the trees of the four runs converged. A pair of gene-specific primers (Additional file 2) was designed for each gene to clone full-length fragments of each of the candidate genes (ranging between 700 and 1400 bp, Additional file 1) except for *Dg*-*gsc* (partial transcript). The cDNA library used was created using a SuperScript™ III First-Strand Synthesis System for RT-PCR kit following RNA extraction with a RiboPure™ Kit and approximately 300 animals (mixed stages and sexes, but mainly adult females). The transcripts were subsequently used to synthesize antisense digoxigenin-labeled RNA probes using an Ambion MEGAscript T7/SP6 Transcription Kit in combination with a TaKaRa RNA in vitro transcription kit. Preliminary studies with antisense and sense probes of other genes indicated a high specificity of the probes (data not shown).

### Whole-mount in situ hybridization (modified from [69])

Fixed, adult female *D. gyrociliatus* were permeabilized with proteinase K (0.005 mg/ml in PTw for 10 min at room temperature) and subsequently treated with glycine (2 mg/ml) and triethanolamine (TEA, 0.1 M pH 7.6; 2 × 5 min; after the first change adding 3 µl/ml acetic anhydride, after five minutes another 6 µl/ml acetic anhydride without changing the TEA solution) to block positive charges. Following washes with 0.1 % PTw, specimens were postfixed in 3.7 % formaldehyde in 0.1 % PTw for 60 min at room temperature, followed by several washes in 0.1 % PTw (5 min each) prior to pre-hybridization and hybridization. These steps were carried out at 62 °C in a hybridization oven. Antisense digoxigenin-labeled riboprobes were diluted to a final concentration of 1 ng/µl in hybridization solution. After hybridization (for 12–36 h at 62 °C), probes were recovered and samples were washed at hybridization temperature with graded series of Hybe buffer to SSC and following to 0.1 % PTw and 0.1 % PBT (PBS + 0.1 % Triton X-100 + 0.1 % BSA (bovine serum albumin)). After two washes in maleic buffer (MAB; 100 mM maleic acid, 150 mM NaCl, 0.1 % Tween-20, pH to 7.5 with NaOH) at room temperature, specimens were blocked for 1 h at room temperature in blocking solution (1 % blocking reagent (Roche)). Antidigoxigenin AP-conjugated antibody (Roche) at a final 1:5000 dilution in blocking solution was incubated overnight at 4 °C. After several washes on 0.1 % PBT followed by 0.1 % PTw at room temperature, samples were transferred to AP buffer and developed with 5-bromo-4-chloro-3-indolyl phosphate 4 toluidine salt (BCIP) and 4-nitro blue tetrazolium (NBT) until the signal was visible. The reaction was stopped in AP buffer without MgCl_2_. Specimens were stored in 70 % glycerol in 0.1 % PTw at 4 °C until further examination.

### Immunohistochemistry after whole-mount in situ hybridization

After the in situ hybridization protocol, adult females of *D. gyrociliatus* were transferred to 0.1 % PTw prior to pre-incubation with 1 % PTA (PBS + 1 % Triton X-100, 0.05 % NaN_3_, 0.25 % BSA and 5 % sucrose). Afterward, samples were incubated for up to 24 h at RT in the primary antibodies monoclonal mouse anti-acetylated α-tubulin in a final concentration of 1:400 in 1 % PBT. Sample specimens for a better description of the nervous system (Fig. 1) were also incubated with polyclonal rabbit anti-serotonin and anti-FMRFamide in a final concentration of 1:200. After several washes in PBS and 1 % PBT, specimens were incubated with the appropriate secondary antibodies conjugated with fluorophores (goat antimouse labeled with CY5, goat antirabbit labeled with TRITC; in a final concentration of 1:400) for up to 48 h at RT. Thereafter, specimens were washed several times in PBS and mounted in fluoromount with DAPI.

### Imaging

**Fig. 2:**
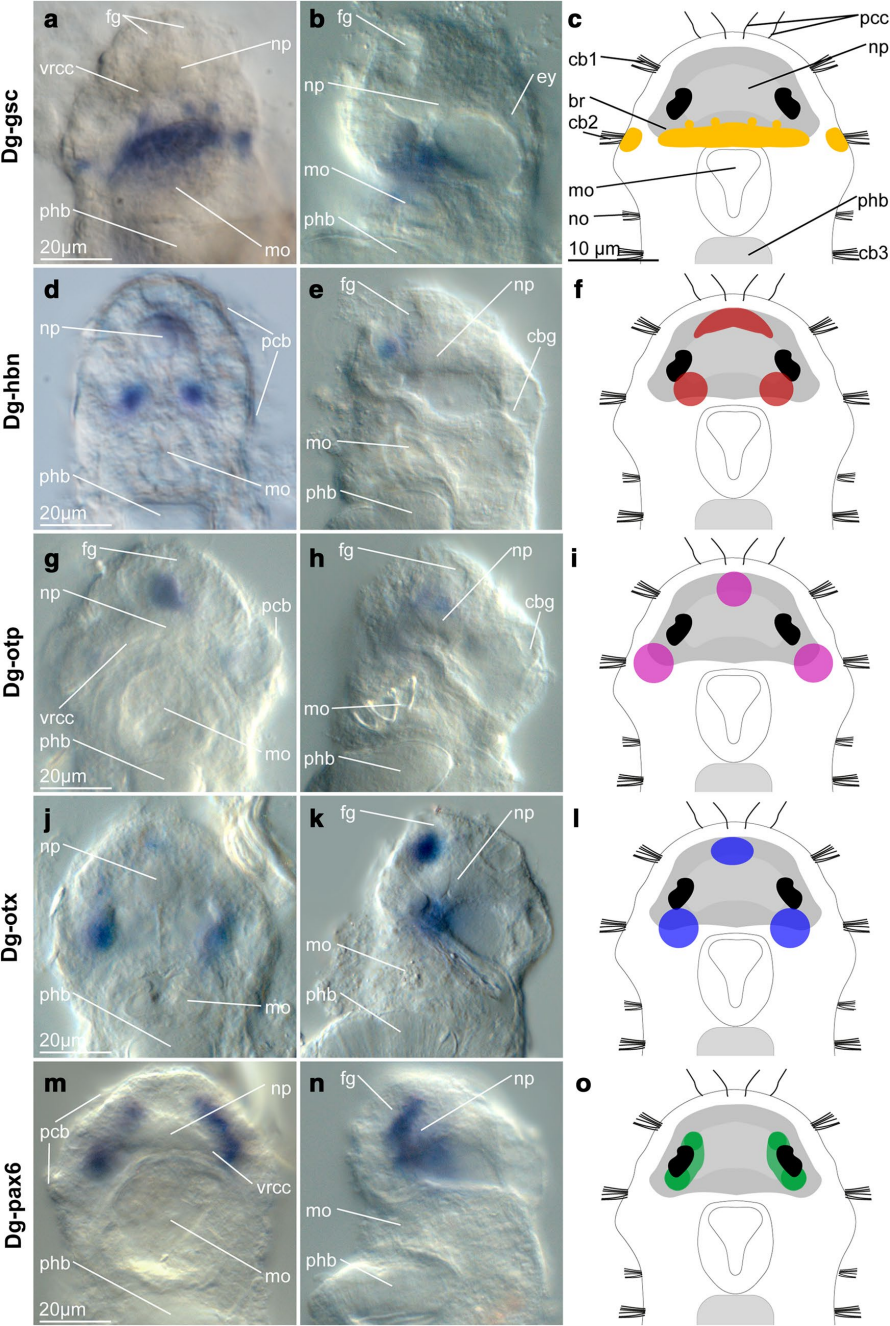
Expression pattern of paired class genes *Dg*-*gsc* (**a**–**c**), *Dg*-*hbn* (**d**–**f**), *Dg*-*otp* (**g**–**i**), *Dg*-*otx* (**j**–**l**) and *Dg*-*pax6* (**m**–**o**) in adult females of *Dinophilus gyrociliatus*. The expression pattern of the respective gene is shown in ventral (**a**, **d**, **g**, **j**, **m**) and lateral (ventral side to the left, **b**, **e**, **h**, **k**, **n**) views as well as in schematic drawings in ventral view (c, f, i, l, o). *Dg*-*gsc* is expressed in the posteroventral region of the brain, the anterior pharyngeal epithelium and two small cell populations close to the second prostomial ciliary band (**a**–**c**). *Dg*-*hbn* shows a spot-like expression, though the individual patches are linked to form a continuous band in the anterior region and two separate posteroventral spots (**d**–**f**). *Dg*-*otp* (**g**–**i**) and *Dg*-*otx* (**j**–**l**) are also expressed in the anterior and posterior region of the brain: *Dg*-*otp* shows a narrow region of expression in the anterior region of the brain (**g**–**i**), while the posterior spots are more clearly demarcated in *Dg*-*otx* (**j**–**l**). *Dg*-*pax6* is found to not only pattern the area of the eyes, but also extend further along the brain surface as well as into the brain (**m**–**o**). *br* brain, *cbg* ciliary band gland, *ey* eye, *fg* frontal gland, *mo* mouth opening, *np* neuropil, *pcb* prostomial ciliary band, *phb* pharyngeal bulb, *vrcc* ventral root of the circumesophageal connective

**Fig. 3:**
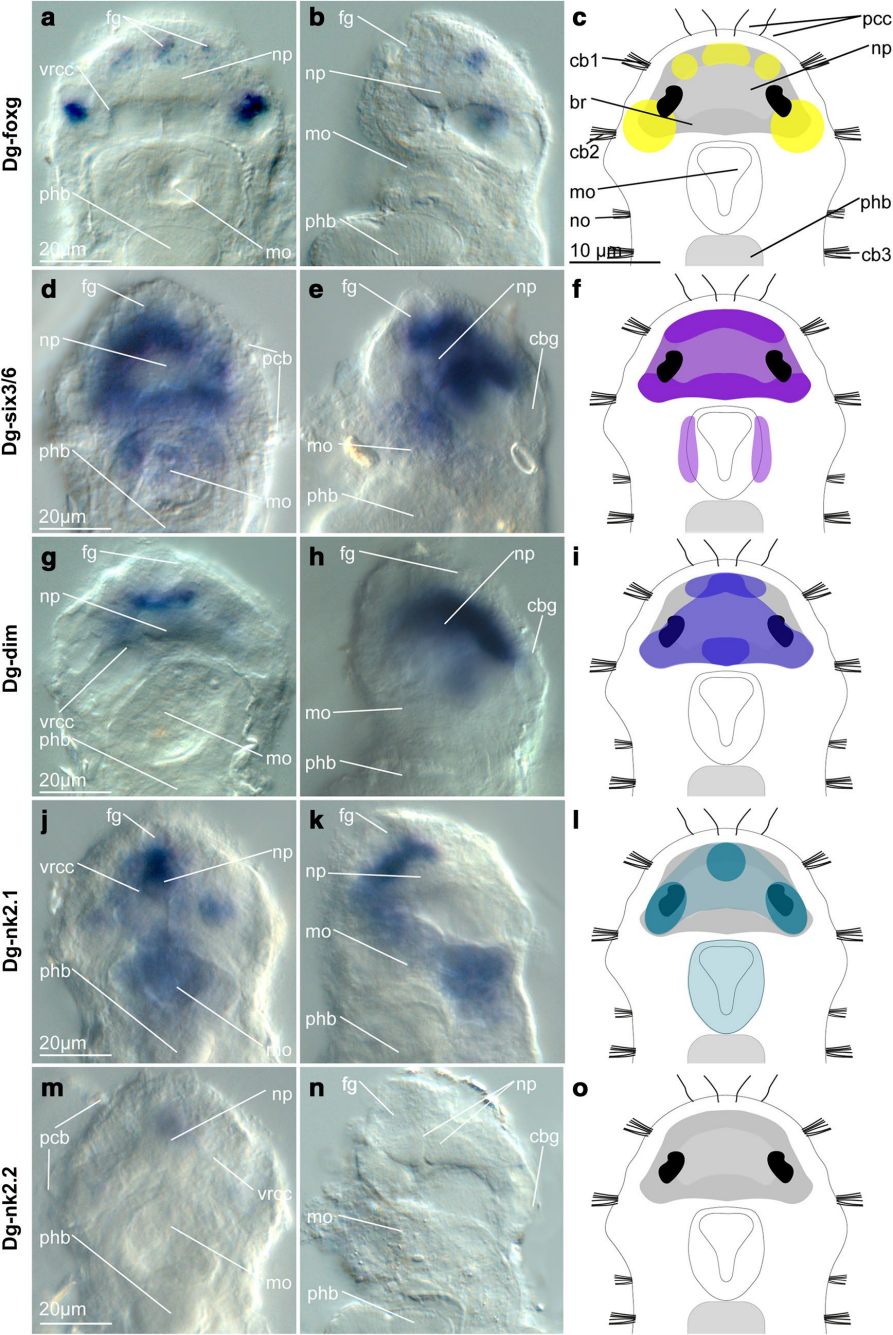
Expression pattern of fox gene *Dg*-*foxg* (**a**–**c**), six class gene *Dg*-*six3*/*6* (**d**–**f**), neuropeptidergic differentiation factor *Dg*-*dim* (**g**–**i**) and NK homeobox genes *Dg*-*nk2.1* (**j**–**l**) and *Dg*-*nk2.2* (**m**–**o**) in adult female *Dinophilus gyrociliatus*. The expression pattern of the respective gene is shown in ventral (**a**, **d**, **g**, **j**, **m**) and lateral (ventral side to the left, **b**, **e**, **h**, **k**, **n**) views as well as in schematic drawings in ventral view (c, f, i, l, o). *Dg*-*foxg* resembles the pattern of *Dg*-*hbn*, but shows more prominent labeling in the anteromedian region and the posterolateral patches, which are shifted anterior to the centers of *Dg*-*hbn* (**a**–**c**). *Dg*-*six3*/*6* labels cells in the entire brain though the ventral side shows gaps between the anterior and posterior continuous patches. Additionally, the anterolateral ectodermal regions of the mouth and pharynx are labeled (**d**–**f**). *Dg*-*dim* shows a complex pattern with a dorsomedian expression, which bifurcates ventrally in the anterior and posterior region, forming a clasp embracing the neuropil (**g**–**i**). *Dg*-*nk2.1* is expressed strongest in the anterior region of the brain and only weakly in the posterior part. However, *Dg*-*nk2.1* also labels the foregut in adult females (**j**–**l**) as well as the hindgut, which is complemented by *Dg*-*nk2.2*, which does not label the brain (**m**–**o**), but the midgut. *br* brain, *cbg* ciliary band gland, *fg* frontal gland, *mo* mouth opening, *np* neuropil, *pcb* prostomial ciliary band, *phb* pharyngeal bulb, *vrcc* ventral root of the circumesophageal connective

**Fig. 4:**
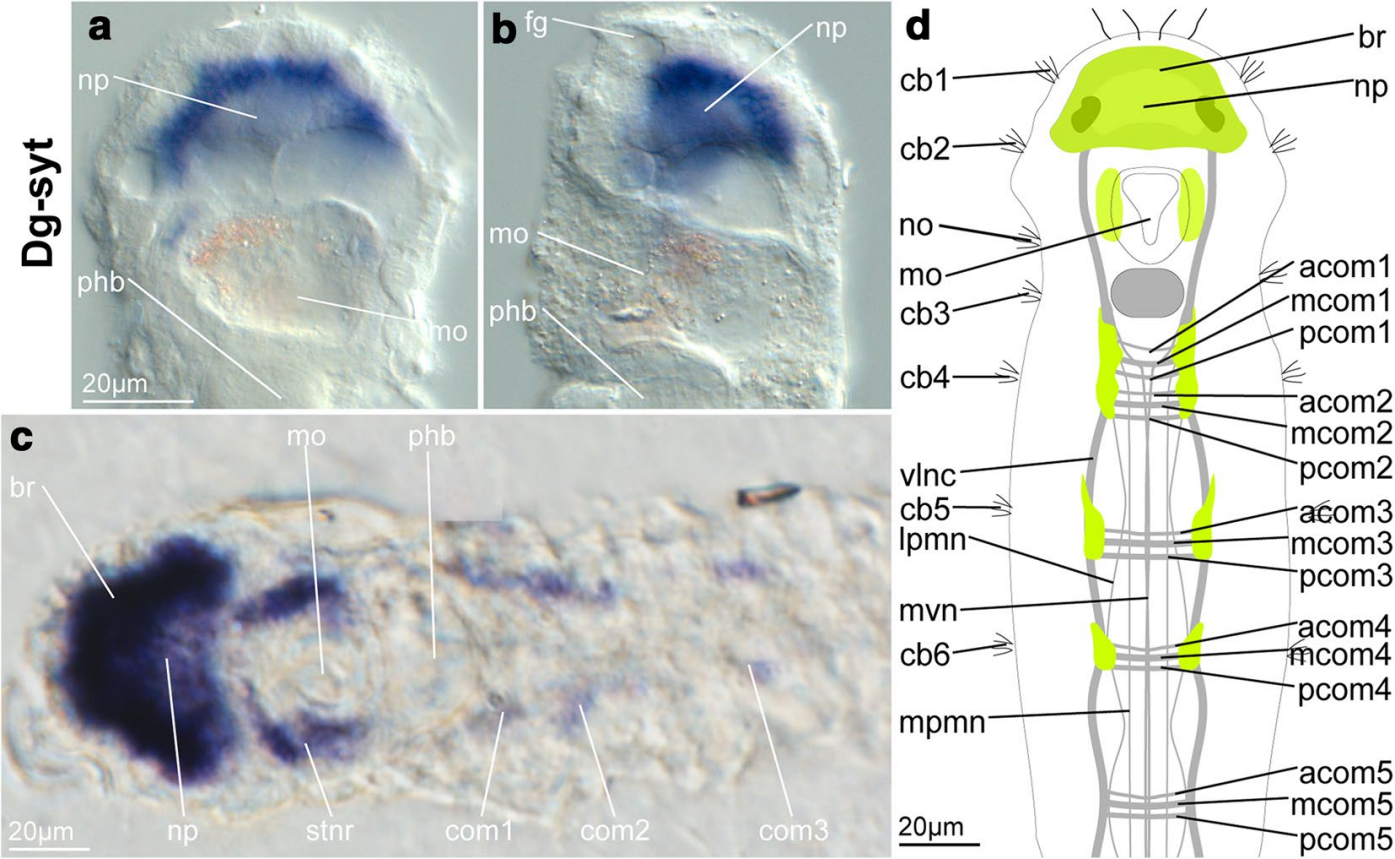
Expression pattern of the membrane-trafficking molecule synaptotagmin-1 *Dg*-*syt* in adult females of *Dinophilus gyrociliatus*. The expression pattern is shown in ventral (**a**, **c**) and lateral (ventral side to the left, **b**) views as well as in a schematic drawing in ventral view (**d**). *Dg*-*syt* labels all perikarya of the brain, thereby resembling the pattern of *Dg*-*six3*/*6*, and can be furthermore shown around the mouth opening and—in approximately 20 % of animals—also labels the ganglionic accumulations in the anterior region of the ventral nerve cord. *acom1*–*4* anterior commissure of segment 1–4, *br* brain, *cb1*–*6* ciliary band 1–6, *com1*–*3* commissural set of segment 1–3, *fg* frontal gland, *lpmn* lateral paramedian nerve, *mcom1*–*4* median commissure of segment 1–4, *mo* mouth opening, *mpmn* median paramedian nerve, *mvn* medioventral nerve, *np* neuropil, *pcom1*–*4* posterior commissure of segment 1–4, *phb* pharyngeal bulb, *stnr* stomatogastric nerve ring, *vlnc* ventrolateral nerve cord

**Fig. 5:**
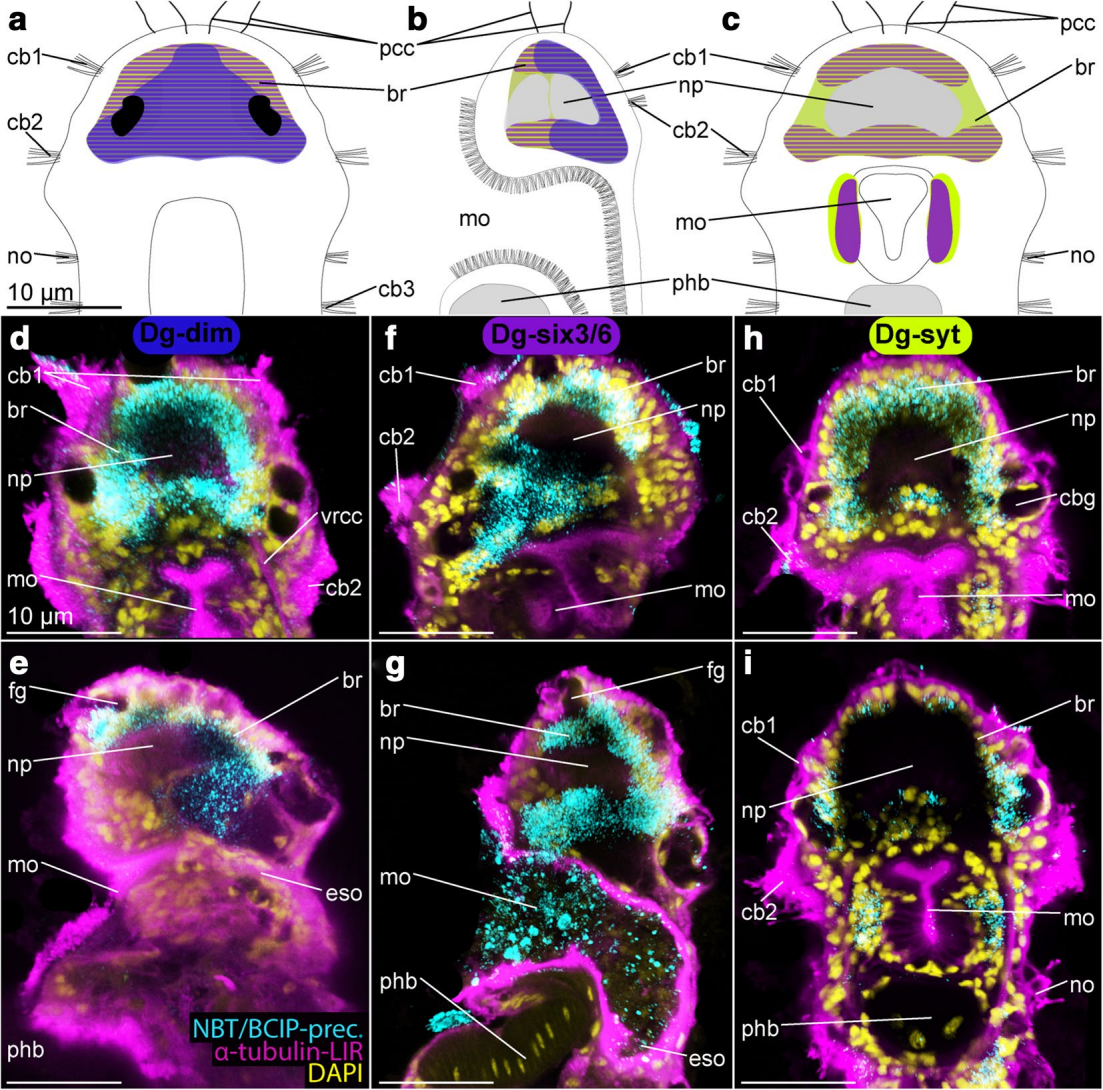
Expression pattern of *Dg*-*dim*, *Dg*-*six3*/*6* and *Dg*-*syt* and in adult females of *Dinophilus gyrociliatus*, which characterize a mediodorsal, probably neurosecretory region of the brain. The assumed overlap of the expression domains is shown in a schematic drawing in dorsal (**a**), lateral (ventral side to the left, **b**) and ventral views (**c**), with similar color coding than employed in the other plates (*Dg*-*dim*—*dark blue*, *Dg*-*six3*/*6*—*purple*, *Dg*-*syt*—*light green*). Reflective microscopy was used to correlate the NBT/BCIP-precipitation pattern (shown in **d**–**i** in *cyan*) with acetylated α-tubulin-like immunoreactivity (acetylated α-tubulin-LIR, *pink*) and DAPI-labeled nuclei (*yellow*). **d** Horizontal section of the ventral region of the brain and **e** horizontal section of the dorsomedian region of the brain with *Dg*-*syt* expression domains, **f** oblique section of the median region of the brain and **g** sagittal section through the brain with *Dg*-*six3*/*6* expression domains, **h** horizontal section of the dorsal region of the brain and **i** sagittal section through the brain with *Dg*-*dim* expression domains. *Scale bar* is 10 µm in all images. *br* brain, *cb* ciliary band, *cbg* ciliary band gland, *eso* esophagus, *fg* frontal gland, *mo* mouth opening, *n* nephridium, *no* nuchal organ, *np* neuropil, *pcb* prostomial ciliary band, *pcc* prostomial compound cilia, *phb* pharyngeal bulb, *vcf* ventral ciliary field, *vrcc* ventral root of the circumesophageal connective

**Fig. 6:**
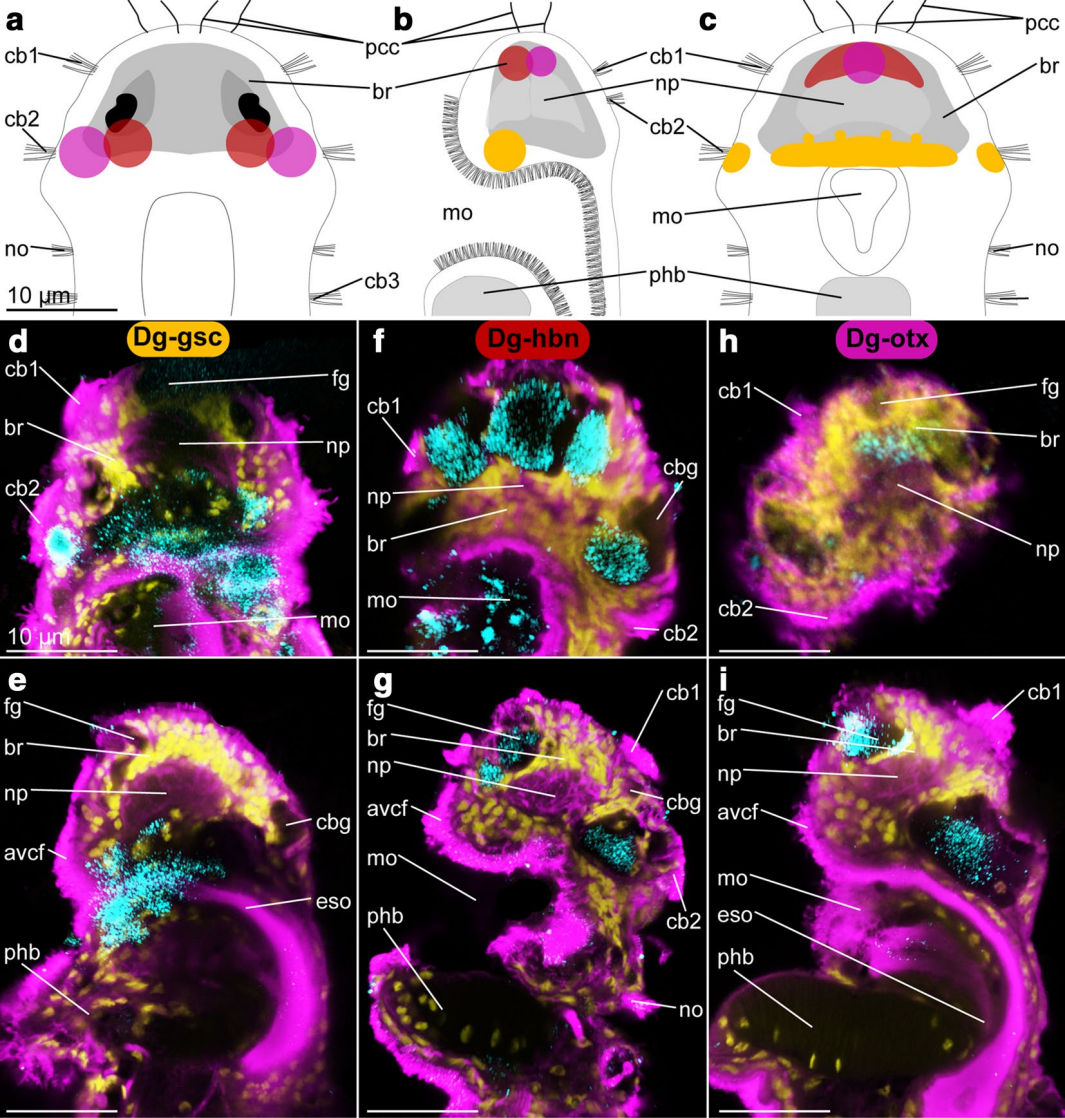
Expression pattern of *Dg*-*gsc*, *Dg*-*hbn* and *Dg*-*otx* in adult females of *Dinophilus gyrociliatus*, which characterize an anteroventral median and posteroventral lateral domain, possibly related to sensory and locomotory cilia. The assumed overlap of the expression domains is shown in a schematic drawing in dorsal (**a**), lateral (ventral side to the left, **b**) and ventral views (**c**), with similar color coding than employed in the other plates (*Dg*-*gsc*—*orange*, *Dg*-*hbn*—*red*, *Dg*-*otx*—*pink*). Reflective microscopy was used to correlate the NBT/BCIP-precipitation pattern (shown in **d**–**i** in *cyan*) with acetylated α-tubulin-like immunoreactivity (acetylated α-tubulin-LIR, *pink*) and DAPI-labeled nuclei (*yellow*). **d** Horizontal section of the ventral region of the brain and **e** sagittal section through the brain with *Dg*-*gsc* expression domains, **f** horizontal section of the ventral region of the brain and **g** sagittal section through the lateral region of the brain with *Dg*-*hbn* expression domains, **h** horizontal section through the dorsal brain and **i** sagittal section through the mediolateral region of the brain with *Dg-otx* expression domains. Scale bar is 10 µm in all images. *avcf* anteroventral ciliary field, *br* brain, *cb* ciliary band, *cbg* ciliary band gland, *eso* esophagus, *fg* frontal gland, *mo* mouth opening, *np* neuropil, *no* nuchal organ, *pcb* prostomial ciliary band, *pcc* prostomial compound cilia, *ph* pharynx, *phb* pharyngeal bulb

**Fig. 7:**
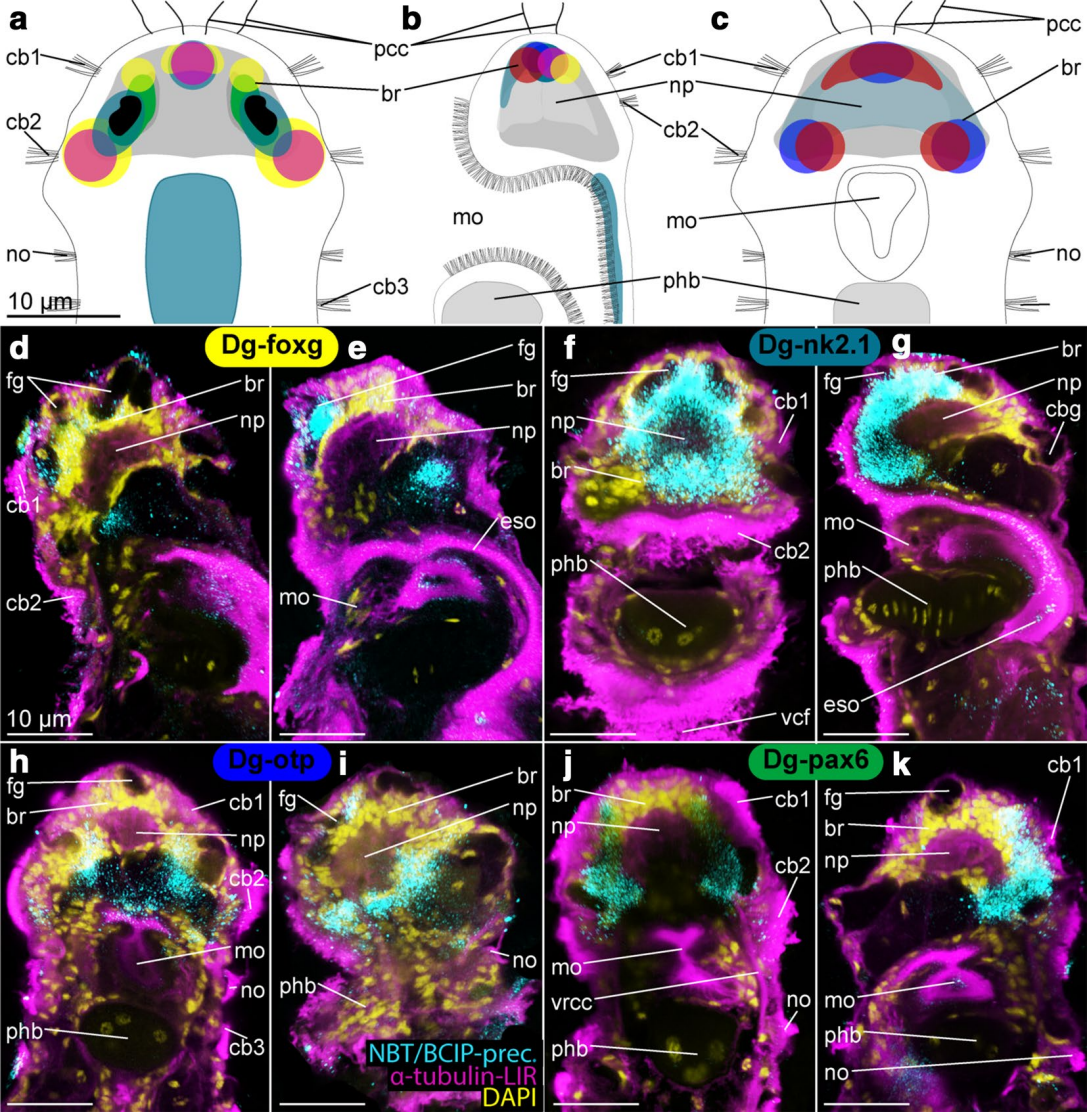
Expression pattern of *Dg*-*foxG*, *Dg*-*nk2.1*, *Dg*-*otp* and *Dg*-*pax6* in adult females of *Dinophilus gyrociliatus*, which are labeling a probable dorsal sensory region including innervation of eyes and sensory cilia (*Dg*-*hbn* and *Dg*-*otx* are included in the schematic drawings, based on information shown in previous plates). The assumed overlap of the expression domains is shown in a schematic drawing in dorsal (**a**), lateral (ventral side to the left, **b**) and ventral views (**c**), with similar color coding than employed in the other plates (*Dg*-*foxg*—*yellow*, *Dg*-*hbn*—*red*, *Dg*-*nk2.1*—*turquoise*, *Dg*-*otp*—*dark purple*, *Dg*-*otx*—*pink*, *Dg*-*pax6*—*dark green*). Reflective microscopy was used to correlate the NBT/BCIP-precipitation pattern (shown in **d**–**k** in *cyan*) with acetylated α-tubulin-like immunoreactivity (acetylated α-tubulin-LIR, *pink*) and DAPI-labeled nuclei (*yellow*). **d** Sagittal section of the lateral region of the brain and **e** sagittal section of the median region of the brain with *Dg*-*foxg* expression domains, **f** horizontal section of the ventral region of the brain and **g** sagittal section through the median region of the brain with *Dg*-*nk2.1* expression domains, **h** horizontal section of the ventral region of the brain and **i** sagittal section through the brain with *Dg*-*otp* expression domains, **j** horizontal section through the dorsal region of the brain and **k** oblique section through the dorsal region of the brain with *Dg*-*pax6* expression domains. *br* brain, *cb* ciliary band, *eso* esophagus, *mo* mouth opening, *no* nuchal organ, *np* neuropil, *pcb* prostomial ciliary bands, *pcc* prostomial compound cilia, *phb* pharyngeal bulb, *vcf* ventral ciliary field

The specimens were mounted in 70 % glycerol in 0.1 % PTw or in Vectashield with DAPI. Images of whole-mount specimens with NBT/BCIP precipitation were taken using a Zeiss Axio Imager 2 Microscope, with mounted Zeiss Axiocam MRc5 camera and the software package Zeiss ZEN 2 or an Olympus IX 70 and a mounted Olympus DP73 microscope camera in combination with the software cellSens (Figs. 2, 3, 4). Immunohistochemically treated samples (Figs. 1, 5, 6, 7) were examined using an Olympus IX 81 inverted microscope with a Fluoview FV-1000 confocal unit. Acquired z-stacks were exported to the Imaris 7.0 software package to conduct further three-dimensional investigations and prepare representative images. Brightness and contrast were adjusted in Adobe Photoshop CC 2015 and plates assembled in Adobe Illustrator CC 2015. The latter also was used to create the schematic drawings.

## Results

### Gene identification and orthology analyses

To gain a better understanding of the molecular regionalization of the brain of the adult female *D. gyrociliatus*, we isolated the transcription factors *dimmed* (*dim*), *foxg*, *goosecoid* (*gsc*), *homeobrain* (*hbn*), *nk2.1*, *nk2.2*, *orthodenticle* (*otx*), *orthopedia* (*otp*), *pax6* and *six3*/*6*, as well as the membrane-trafficking protein *synaptotagmin*-*1* (*syt*) by gene-specific PCR (Additional file 1). We performed Bayesian analysis to confirm their orthology (Additional files 1, 3, 4, 5, 6, 7, 8) and referred to *D. gyrociliatus* orthologous genes as *Dg*-*dim*, *Dg*-*foxg*, *Dg*-*gsc*, *Dg*-*hbn*, *Dg*-*nk2.1*, *Dg*-*nk2.2*, *Dg*-*otx*, *Dg*-*otp*, *Dg*-*pax6*, *Dg*-*six3*/*6* and *Dg*-*syt*.

### Expression of the neural PRD class genes *Dg*-*gsc*, *Dg*-*hbn*, *Dg*-*otp*, *Dg*-*otx* and *Dg*-*pax6*

**Fig. 8:**
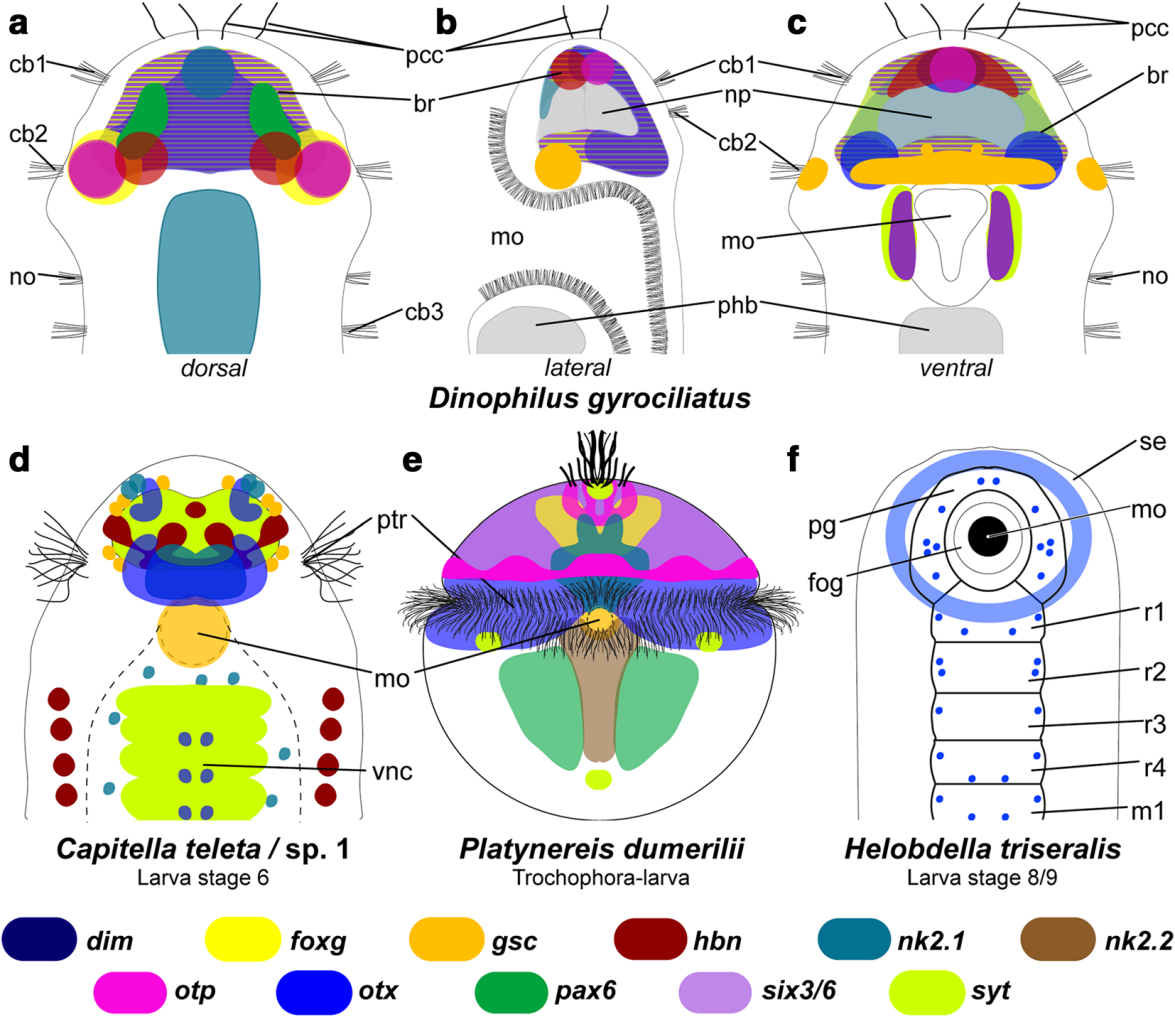
Axial patterning in the brain of adult females of *Dinophilus gyrociliatus* and comparison of orthologous gene expression in stage 8 larva of *Capitella teleta*, a trochophore larva of *Platynereis dumerilii* and a stage 8/9 larva of *Helobdella triseralis*. **a**–**c** Schematic interpretation based on single-probe NBT/BCIP-precipitation patterns acquired in this study (*Dg*-*dim*, *Dg*-*foxG*, *Dg*-*gsc*, *Dg*-*hbn*, *Dg*-*nk2.1, Dg*-*otp*, *Dg*-*otx*, *Dg*-*pax6, Dg*-*six3*/*6* and *Dg*-*syt*) in dorsal (**a**), midsagittal (ventral side to the left, **b**) and ventral (**c**) views. **d** Ventral view of the trochophore larva in *P. dumerilii* inferred from [23, 35, 40], **e** ventral view of the anterior region in *C. teleta* inferred from [16, 33], **f** ventral view of embryonic stage 8/9 in *Helobdella triseralis* [41]. The overlap of genes is interpreted from this and previous studies. *fog* foregut, *mo* mouth opening, *m1* segmental midbody ganglion, *np* neuropil, *pcc* prostomial compound cilia, *pcb* prostomial ciliary bands, *pg* prostomial ganglion, *ptr* prototroch, *r1–r4* segmental rostral ganglion, *se* surface ectoderm, *vnc* ventral nerve cord

The transcription factor *goosecoid* (*Dg*-*gsc*) was expressed in a narrow region in the posteroventral brain (Fig. 2a–e) and between this region and the anterior region of the pharyngeal epithelium (Figs. 2a–c, 6d, e, 8b, c). Additionally, four distinct cells (or small groups of cells) were labeled anteriorly to this broad domain in the brain (Figs. 2a–c, 8c). Reflective microscopy further suggested that this gene is also expressed lateral in the prostomium, adjacent to the second ciliary band, where these domains extend further ventrally and dorsally than the expression region of the brain (Figs. 6d, e, 8b).

The gene *homeobrain* (*Dg*-*hbn*) showed a much weaker expression in the anterodorsal region of the brain (Figs. 2d–f, 6f, g, 8b, c) and two lateral spots with stronger expression, where the ventral root of the circumesophageal connective extends posteriorly from the neuropil and brain (Figs. 2d, f, 7f, g, 8a).

The transcription factor *orthopedia* (*Dg*-*otp*) had a very narrow expression pattern (Figs. 2g, i, 7h, i, 8a–c), which consisted of a strongly labeled spot in the anteroventral region of the brain (Figs. 2g–i, 7i, 8b, c), between the nerves extending to the prostomial compound cilia, and a pair of less prominent, but more extended patches ventral to the eyes, dorsal to the neuropil (Figs. 2g–i, 7h, i, 8a). This pattern superficially resembled the one found with *orthodenticle* (*Dg*-*otx*). However, *Dg*-*otx* was expressed more ventrally in the posterior part of the brain (Figs. 2j–l, 6i, 8b, c), in one pair of patches at the base of the ventral root of the circumesophageal connective (Figs. 2j–l, 6i, 8b). The anterior expression pattern of *Dg*-*otx* resembles the anterior domain of *Dg*-*hbn*, although it is located more ventrally and was weaker than the other studied domains (Figs. 2j–l, 6i, 8c).

Finally, the gene *pax6* (*Dg*-*pax6*) was detected in one pair of lateral elongated domains, roughly ventral to the eyes (Figs. 2m, o, 7j, k, 8a). However, the expression was deeper within the brain and not only on the dorsal surface, where the eyes locate (Figs. 2n, o, 7j).

### Expression of the Fox class gene *Dg*-*foxg*

The *forkhead transcription factor G* (*Dg*-*foxg*) was expressed in one pair of strongly labeled posteromedial patches close to the ventral root of the circumesophageal connective (Figs. 3a–c, 7d, e, 8a). Additionally, a series of small dot-like patches formed a sickle-shaped, narrow line in the anteromedian region of the brain, approximately spanning between the nerves innervating the anterior prostomial ciliary band (Figs. 3a–c, 7d, e).

### Expression of the six class gene *Dg*-*six3*/*6*

The six class gene *six3*/*6* (*Dg*-*six3*/*6*) had a rather broad expression pattern (Figs. 3d–f, 5f, g, 8a–c) when compared to the genes described above (Figs. 2, 6, 7, 8a–c). *Dg*-*six3*/*6* showed a nearly continuous dorsoventral pattern anterior and posterior to the neuropil, which was connected by strongly labeled lateral regions, forming a dorsolateral clasp around the neuropil (Figs. 3e, f, 5f, g, 8a–c). On the ventral side, only the transverse patches anterior and posterior to the neuropil were detected (Figs. 3d, f, 5g). Additionally, a pair of elongated, bean-shaped expression domains localized at the anterolateral edge of the mouth (Figs. 3d, f, 5f, g, 8c).

### Expression of the neurosecretory differentiation factor *Dg*-*dim*

The gene *dimmed* is a neurosecretory differentiation factor present in the apical organ of *P. dumerilii* [70] and in specific neural populations in the brain and ventral nerve cord in the larvae and adult of *D. melanogaster* [71–73]. In adult *D. gyrociliatus* females, *Dg*-*dim* showed a mainly dorsal pattern in the brain, which is broad in the anterior and posterior region of the brain, but more narrow on the dorsal side than, e.g., the domains formed by *Dg*-*six3*/*6* (Figs. 3g–i, 5d, e).

### Expression of the NK homeobox genes *Dg*-*nk2.1* and *Dg*-*nk2.2*

Different to other brain markers, such as *Dg*-*six3*/*6* and *Dg*-*dim*, the transcription factor *Dg*-*nk2.1* exhibited a stronger expression on the anteroventral and posterodorsal side of the neuropil (Figs. 3j–l, 7f, g, 8b, c). Additionally, a pair of spot-like domains was located laterally to the eyes and medioventrally to the neuropil (Figs. 3j, l. 7g). The three anterior patches (ventromedian and mediolateral) were connected by a broader domain ventral to the neuropil (Figs. 3j–l, 7f, g, 8a, b). The dorsal region of the brain did not show any additional labeling with *Dg*-*nk2.1* (Figs. 3j–l, 7g, 8a). Additionally, *Dg*-*nk2.1* is also expressed in the pharyngeal epithelium (Figs. 3j–l, 7f). We did not detect any expression of *Dg*-*nk2.2* in the brain (Fig. 3m–o), but in the mid- and hindgut (Additional file 9).

### Expression of *Dg*-*syt*

The gene *synaptotagmin*-*1* (*Dg*-*syt*) got recognized recently as a broad nervous system marker [33]. In accordance with other annelids such as *C. teleta* [33] and *P. dumerilii* [35], which express *synaptotagmin*-*1* in both the brain and the ventral nerve cords, we detected expression of *Dg*-*syt* in the majority of perikarya of the brain in females of *D. gyrociliatus* (Figs. 4a–d, 5h, 8a–c) and in ganglionic accumulations along the anterior ventral nerve cord in about 20 % of all investigated specimens (Fig. 4c), which is possibly due to the small amount and weak concentration of cell nuclei at the commissures, in particular when compared to, e.g., *C. teleta* [33]. It was observed that these cellular accumulations associated with the commissures are strongest in young females and get more dilated in older/pregnant specimens.

## Discussion

### Molecular patterning of the brain in adult females of *D. gyrociliatus*

Nine out of the eleven evolutionarily conserved neural genes analyzed in this study show domains mainly within or closely adjacent to the brain (*Dg*-*dim*, *Dg*-*foxg*, *Dg*-*gsc*, *Dg*-*hbn*, *Dg*-*otp*, *Dg*-*otx*, *Dg*-*pax6*, *Dg*-*six3*/*6* and *Dg*-*syt*, Figs. 1, 2, 3, 4, 5, 6, 7, 8; Additional file 9). Exceptions are transcription factors *Dg*-*nk2.1*, expressed in the fore- and hindgut in addition to the domains in the brain (Figs. 3j–l, 8a–c; Additional file 9), and *Dg*-*nk2.2*, not detected in the brain, but in the mid- and hindgut (Fig. 3m–o; Additional file 9).

*Dg*-*gsc* is not exclusively expressed in the brain, but also in two condensed domains close to the second prostomial ciliary band (Figs. 2a–c, 6d, e, 8c), which are associated with neither the brain nor the digestive tract. Clearly demarcated domains resembling this pattern cannot be found in either *C. teleta* or *P. dumerilii* [74, 75]. Although *goosecoid* domains in the stomodeum and foregut region are present in the trochophore larva of *P. dumerilii* (Fig. 8e, [75]) as well as the early larva of *C. teleta* (Fig. 8d, [74]), they are limited to the anterior portion of the pharyngeal epithelium in adult female *D. gyrociliatus* (Figs. 2a–c, 6d, e, 8b, c; Additional file 9). The *gsc*-positive domains within the brain vary between the investigated annelids. They are restricted to the foregut in trochophore larvae of *P. dumerilii* (Fig. 8e, [75]), but localized in the posterior part of the brain, lateral to the brain and in the region of the circumesophageal connectives in late larvae of *C. teleta* (larval stage 7–8, Fig. 8d, [74]) and adult *D. gyrociliatus* (Figs. 2a–c, 8b, c; Additional file 9). Our findings thereby suggest that *goosecoid* can vary in its expression in the brain and stomatogastric system in Annelida.

*Dg*-*hbn* is expressed in distinctive parts of the brain in adult females of *D. gyrociliatus* (Figs. 2d–f, 6f, g, 8a–d), as also observed in *C. teleta* [16], with several patches in the posterior part of the brain extending to the anterior (mainly in the latter). Additionally, segmentally arranged domains of *CapI*-*hbnl* in *C. teleta* were detected in association with chaetal sacs lateral along the body length [16], which were not found in *D. gyrociliatus* females. *Homeobrain* is therefore probably involved in neuronal differentiation in the cerebral ganglia and brain in annelids.

*Dg*-*otp* is expressed both in a median, demarcated domain on the ventroanterior side and one pair of lateral patches in the ventroposterior region of the brain in *D. gyrociliatus* (Figs. 2g–i, 7h, i, 8a–c; Additional File 9). The anterior domain seems to occur within a *Dg*-*six3*/*6*-positive region of the brain, which resembles the situation found in invertebrate apical organs and especially in the apical organ of *P. dumerilii*, where *orthopedia* is present in presumptive serotonergic cells [22, 23]. In adult females of *D. gyrociliatus*, however, *Dg*-*otp* is not related to any serotonergic component, since these are restricted to the dorsoposterior region of the brain (pers. obs., Fig. 1g, [45]). More posteriorly, *otp* is expressed in cells that might be involved in forming the multiciliated cells of the prototroch in the trochophore larvae of *P. dumerilii* [23]. Although information about its expression in *C. teleta* is missing, the expression of *otp* in cells related to several cells of the second prostomial ciliary band in *D. gyrociliatus* (Figs. 2g–i, 8a–c) is thus probably an indication for the remnants of the prototroch in the later.

*Dg*-*otx* is expressed broadly as continuous band in the ventroanterior region of the brain as well as in one pair of lateral domains in the posterior region of the brain. It is therefore overlapping with *Dg*-*six3*/*6* in female *D. gyrociliatus* in both the anterior and the posterior part of the brain (Figs. 2g–i, 8a–c). This is in contrast to the expression detected in *P. dumerilii* trochophore larvae (Fig. 8e), where *otx* demarcates the ventral expression range of *six3*/*6* posterior to the cells expressing the PRD class gene *otp*, tracing the prototroch [21, 23, 34]. It is only partly in concordance with the pattern of the transcription factor *otx* in *C. teleta*, which develops domains anterolateral and posteromedian in the brain, but also in the foregut, posterior region of the midgut, hindgut and the medioventral ectoderm [74]. The expression domains of the PRD class gene *ot*x are thus little conserved among the anterior neural regions in the annelid species analyzed so far. Further studies have to ascertain whether the exclusiveness of *six3*/*6* and *otx* as observed in the apical organ and the posterior region of the brain in *P. dumerilii* trochophore larvae [21, 34] or the broad expression in the brain and digestive system as detected in *C. teleta* [74] are also present in the meiofaunal annelid during earlier developmental stages. The differences among the three annelids thereby might be the result of temporal variability of this gene’s activation rather than an expression in different domains.

*Dg*-*pax6* is expressed in extended patches in the regions of the eyes dorsal to the neuropil in the brain of *D. gyrociliatus* (Figs. 2m–o, 8a–c). This matches the transcription factor’s proposed role in eye development across Bilateria [40, 76], based on its bilateral brain domains, being the most common location of photoreceptors [9, 35]. The lateral patterns found in the median coronal plane of the brain of *D. gyrociliatus* might also indicate that it is involved in specifying the mediolateral axis of the central nervous system as it has been suggested for the bilaterian ancestor [35].

*Dg*-*foxg* is expressed in domains in the anterior and the posterior region of the brain, which resembles the pattern detected with *Dg*-*otp* and *Dg*-*otx* (Figs. 2g–l, 3a–c, 8a–c). The anterior domain is constituted by an interconnected series of spots, while there is a pair of dorsolateral demarcated domains in the posterior part of the brain (Figs. 3a–c, 8a–c). This seems to be contradicting the finding in the trochophore larvae of *P. dumerilii*, where this gene is related to the developing cerebral ganglia and especially the mushroom bodies [9], and thereby labels more central neural structures (Fig. 8e). The different patterns in the annelids studied so far might either be related to the developmental stage the animals are in (trochophore larvae in *P. dumerilii* vs. adults in *D. gyrociliatus*) or to the different organization of the nervous system (compartmentalized in *P. dumerilii* vs. compact and seemingly unstructured in *D. gyrociliatus*). Further information in *C. teleta* (adult and developmental stages), which has an unstructured brain, but indirect development, will aid the reconstruction of a general annelid pattern.

All perikarya of the brain with the exception of the ventrolateral sides also express *Dg*-*six3*/*6* (Figs. 3d–f, 5f, g, 8a–c; Additional file 9), which therefore shows broad overlap with the other genes used in this study (Fig. 8a–c). This supports previous findings about *six3*/*6* playing a major role in demarcating the anterior or anteromedian region of the brain in several invertebrate groups [22, 34, 77], and especially in patterning the larval apical organ and cerebral ganglia in the annelids *C. teleta* and *P. dumerilii* (Fig. 8d, e, [23]).

The most elaborate dorsal pattern is formed by *Dg*-*dim*, which is strongest along the dorsal midline while expanding antero- and posteromedially in the brain (Figs. 3g–i, 5d, e, 8a–c; Additional file 9). The transcription factor *dimmed*, which is suggested to be involved in patterning neurosecretory cells during early and larval development, is detected in several cells of both the brain and the ventral nerve cord in the ecdysozoan *D. melanogaster* [71, 72] and in few cells in the apical organ of the trochophore larva of the annelid *P. dumerilii* [70]. In *Drosophila*, the protein DIMMED directly activates the neuropeptide-amidating enzyme PHM [72]. Since amidated neuropeptides are generally conserved between related animal lineages, e.g., spiralian groups [78], further work in more annelid species is essential to better understand the evolution of *dimmed*-positive neurons.

*Dg*-*nk2.1* and *Dg*-*nk2.2* do not exclusively pattern the brain, since *Dg*-*nk2.1* is also expressed in the roof of the foregut and the entire hindgut (Figs. 3j–l, 7f, g, 8a–c; Additional file 9), and *Dg*-*nk2.2* labels the midgut and is completely absent from the adult brain of the female *D. gyrociliatus* (Fig. 3m–o; Additional file 9). *Capitella teleta* shows similar anterolateral (and posteromedian) expression domains of *nk2.1* in the brain, which are supplemented by strongly labeled regions in the fore- and hindgut, as well as spot-like expression in the remaining digestive tract (Fig. 8d (domain in the foregut shadowed by broader region of *otx*), [74]). In *P. dumerilii*, *nk2.1* was found associated with the mouth opening and the ventral side of the trochophore larva, where it extends from approximately the apical organ (where it has a broad expression domain) toward the mouth opening, overlapping with *six3*/*6* (Fig. 8e, [18, 22, 23]). This latter somewhat discrepant pattern emphasizes the need for developmental studies of *D. gyrociliatus* for proper comparison, but the overall resemblance among all hitherto studied annelids supports a probable function of the transcription factor *nk2.1* in differentiating the ventral region of the brain as well as the digestive system in annelids.

The transcription factor *nk2.2* strictly complements the pattern of *pax6* in the developing nervous system in the trochophore larva of *P. dumerilii* (Fig. 8e, [35]). It does not have domains anterior to the prototroch, but is expressed in a slender region of midline cells, thereby labeling the medial edges of the fusing neuroectoderm and possibly lining the prototroch lateral to the stomodeum (Fig. 8e, [35]). In later stages, the domain is limited to the midventral region between the prominent nerve cords [35]. Although we did not observe expression of *Dg*-*nk2.2* in the brain in adult *D. gyrociliatus*, further expression analyses in juveniles and embryonic stages will help uncover possible neural-related expression of *nk2.2* in this annelid species.

Adult female *D. gyrociliatus* show expression of *Dg*-*syt* in the brain, but also in the ganglionic accumulations along the ventral nerve cord (associated with the commissural sets, Figs. 4c, d, 8a–c), which is similar to previous findings in other annelids such as *C. teleta* and *P. dumerilii* [33, 35]. S*ynaptotagmin*-*1* is expressed in individual cells of the apical organ or early adult brain and along the ventral nerve cords, seemingly involved in the formation of the ganglionic cord of *C. teleta* and *P. dumerilii* [33, 35]. We therefore suggest that *Dg*-*syt* plays a similar role in neural specification in *D. gyrociliatus*. [29, 31].

Altogether, our findings indicate that the overall patterning of the brain in adult females of *D. gyrociliatus* resembles the molecular regionalization observed in larval stages of previously examined annelids (e.g., [16, 23, 37, 40, 79, 80]). We furthermore identified that (1) *Dg*-*dim*, *Dg*-*six3*/*6* and *Dg*-*syt* pattern a mediodorsal, probably neurosecretory region, which also extends ventrally anterior and posterior to the neuropil (Fig. 5), (2) *Dg*-*foxg*, *Dg*-*gsc* and *Dg*-*otx* demarcate a population of cells underneath the second ciliary band of the prostomium (Fig. 6), and (3) *Dg*-*foxg*, *Dg*-*hbn*, *Dg*-*otp*, *Dg*-*otx*, *Dg*-*nk2.1* and *Dg*-*pax6* specify sensory regions in the brain such as the anterior neural region (probably related to the prostomial compound cilia), the eyes and the base of the nerves extending from the nuchal organs to the neuropil (Fig. 7). However, more detailed investigations of the embryonic and juvenile stages in *D. gyrociliatus* will provide a better insight into the role of these genes during neural development.

### Miniaturization and the molecular patterning of the brain in Annelida

Meiofaunal species and their organ systems have not been included in previous studies of annelid and spiralian diversity although their inclusion will likely provide additional insight into how conserved molecular networks are modified to give rise to differently organized tissues. In this context, it is still unclear whether neural genes, which are broadly conserved in macroscopic representatives of Protostomia and Deuterostomia [52, 53, 81, 82, 83], maintain their relative expression domains in brains of microscopic size and limited cell number, as those found in interstitial animals. Our findings demonstrate that the relative position of genes such as *foxg*, *orthopedia*, *homeobrain*, *six3*/*6* and *synaptotagmin*-*1* seems to be similar between *D. gyrociliatus* and other annelids and—to some extent—Spiralia (Figs. 1, 2, 3, 4, [16, 23, 33, 35]), although the approximate number of cells in the respective domains is lower. Importantly, these observations uncover underlying substructures in the small, compact brain of *D. gyrociliatus*. The fact that we observed similar molecular domains in *D. gyrociliatus* and other annelid taxa suggests that the relative extent of the expression areas is maintained regardless of the life stage of the animal. This furthermore suggests that the cells in the brain of *D. gyrociliatus* do not show a higher degree of multifunctionality, but that in these microscopic brains probably fewer cells are assigned to certain functions. Further studies unraveling the earlier developmental stages of *D. gyrociliatus* as well as the dwarf male with an even smaller brain consisting of only 42 cells [63, 64] are therefore highly warranted. They will not only help to generate a broader base for comparisons between annelid and/or spiralian taxa, but also deepen our understanding of the conservation of genetic patterning of the brain in species with different neuroanatomies adapted to particular ecological niches and possibly varying requirements in different life stages (encapsulated embryos, free-swimming larvae, adults).

## Conclusions

Adult females of *D. gyrociliatus* express *dim*, *foxg*, *gsc*, *hbn*, *otp*, *otx*, *nk2.1*, *pax6*, *six3*/*6* and *syt* almost exclusively in the brain, and their pattern is consistent with the domains described in larval stages in the macrobenthic annelid species *P. dumerilii* and *C. teleta* (e.g., [16, 21, 23]). Although the brain does not show elaborate morphological substructures (e.g., mushroom bodies, optic lobes/tracts), *PRD box* and *FOX* genes and *nk2.1* and the transcription factor *dimmed* are expressed in specific areas in the brain, with moderate overlap between their patterns. We therefore suggest that the adult brain in this meiofaunal annelid is also regionalized and possibly shows more similarities with late larval stages in *C. teleta* [16, 33] than suggested by gross morphology alone [45, 46]. The overall expression pattern in the anterior nervous system as described previously in several annelid species [22, 23] is also observed in the adult brain of *D. gyrociliatus*, despite the interspecific differences in the organ size and developmental mode. Therefore, we propose that the underlying patterning mechanism of the brain is independent of whether the animals show direct or indirect development and of the final complexity of this anterior neural region. To what extend the molecular regionalization of the brain can be correlated with different functions of the respective areas has to be tested in future studies, also including earlier developmental stages.

## Additional files

10.1186/s13227-016-0058-2 Sequences for gene orthology assignment. Amino acid sequences for *Dinophilus gyrociliatus* genes used in this study as well as related proteins from other animals, retrieved from NCBI (html://http://ncbi.nlm.nih.gov) and Joint Genome Institute (http://genome.jgi-psf.org/Capca1/Capca1.home.html for *Capitella teleta*; http://genome.jgi-psf.org/Lotgi1/Lotgi1.home.html for *Lottia gigantea*; http://genome.jgi-psf.org/Nemve1/Nemve1.home.html for *Nematostella vectensis*; http://genome.jgi-psf.org/Triad1/Triad1.home.html for *Trichoplax adhaerens*) including all information for the Additional files 3, 4, 5, 6, 7 and 8. http://genome.jgi-psf.org/Capca1/Capca1.home.html for *Capitella teleta*; for *Lottia gigantea*; for *Nematostella vectensis*; for *Trichoplax adhaerens*) including all information for the Additional files 3, 4, 5, 6, 7 and 8.
10.1186/s13227-016-0058-2 Primer sequences used for the respective genes in *Dinophilus gyrociliatus.* Matching primer pairs were designed using MacVector (MACVECTOR, INC., Cambridge, UK) and the “*Sequencing Primers*/*Probes*”-tool. Pairs were chosen based on their length (20–25 bp), G-C-content (45–55 %, manually checked for not more than 3 C or G in a row) and similar working temperature.
10.1186/s13227-016-0058-2 Phylogenetic analysis of *Dg*-*syt*. Phylogenetic tree of the membrane-trafficking protein SYNAPTOTAGMIN-1 with emphasis on Dg-syt (which is highlighted in the tree), supporting its orthology assignment. Protein alignments were made using MUSCLE [67] and Bayesian phylogenetic analysis was performed using MrBayes [68], with settings according to [22]. Each analysis was run for 30,000,000 generations sampled every 1000 generations in four runs. A consensus tree and posterior probabilities for each branch were calculated prior to visualization of the tree with FigTree and edition in Adobe Illustrator 2015CC. All sequences used are listed in Additional file 1.
10.1186/s13227-016-0058-2 Phylogenetic analysis of PRD class genes *Dg*-*gsc*, *Dg*-*hbn*, *Dg*-*otp*, *Dg*-*otx* and *Dg*-*pax6*. Phylogenetic tree of the paired box genes with emphasis on the genes used in this study (which are highlighted in the tree), supporting their orthology assignments. Protein alignments were made using MUSCLE [67] and Bayesian phylogenetic analysis was performed using MrBayes [68], with settings according to [22]. Each analysis was run for 30,000,000 generations sampled every 1000 generations in four runs. A consensus tree and posterior probabilities for each branch were calculated prior to visualization of the tree with FigTree and edition in Adobe Illustrator 2015CC. All sequences used are listed in Additional file 2.
10.1186/s13227-016-0058-2 Phylogenetic analysis of *Dg*-*foxG*. Phylogenetic tree of the forkhead box genes with emphasis on *Dg*-*foxG* (which is highlighted in the tree), supporting its orthology assignments. Protein alignments were made using MUSCLE [67] and Bayesian phylogenetic analysis was performed using MrBayes [68], with settings according to [22]. Each analysis was run for 30,000,000 generations sampled every 1000 generations in four runs. A consensus tree and posterior probabilities for each branch were calculated prior to visualization of the tree with FigTree and edition in Adobe Illustrator 2015CC. All sequences used are listed in Additional file 1.
10.1186/s13227-016-0058-2 Phylogenetic analysis of *Dg*-*six3*/*6*. Phylogenetic tree of the six class genes with emphasis on *Dg*-*six3*/*6* (which is highlighted in the tree), supporting its orthology assignments. Protein alignments were made using MUSCLE [67] and Bayesian phylogenetic analysis was performed using MrBayes [68], with settings according to [22]. Each analysis was run for 30,000,000 generations sampled every 1000 generations in four runs. A consensus tree and posterior probabilities for each branch were calculated prior to visualization of the tree with FigTree and edition in Adobe Illustrator 2015CC. All sequences used are listed in Additional file 1.
10.1186/s13227-016-0058-2 Phylogenetic analysis of *Dg*-*dim*. Phylogenetic tree of the dimmed genes with emphasis on *Dg*-*dim* (which is highlighted in the tree), supporting its orthology assignments. Protein alignments were made using MUSCLE [67] and Bayesian phylogenetic analysis was performed using MrBayes [68], with settings according to [22]. Each analysis was run for 30,000,000 generations sampled every 1000 generations in four runs. A consensus tree and posterior probabilities for each branch were calculated prior to visualization of the tree with FigTree and edition in Adobe Illustrator 2015CC. All sequences used are listed in Additional file 1.
10.1186/s13227-016-0058-2 Phylogenetic analysis of *Dg*-*nk2.1* and *Dg*-*nk2.2*. Phylogenetic tree of the NK-homeobox genes with emphasis on the genes used in this study (which are highlighted in the tree), supporting their orthology assignments. Protein alignments were made using MUSCLE [67] and Bayesian phylogenetic analysis was performed using MrBayes [68], with settings according to [22]. Each analysis was run for 30,000,000 generations sampled every 1000 generations in four runs. A consensus tree and posterior probabilities for each branch were calculated prior to visualization of the tree with FigTree and edition in Adobe Illustrator 2015CC. All sequences used are listed in Additional file 1.
10.1186/s13227-016-0058-2 Brain-specificity of gene expression patterns for the genes tested. *Dg*-*syt*, *Dg*-*gsc*, *Dg*-*hbn*, *Dg*-*otp*, *Dg*-*otx*, *Dg*-*pax6*, *Dg*-*foxG*, *Dg*-*six3*/*6* and *Dg*-*dim*, while *Dg*-*nk2.1* also labels the fore- and hindgut and *Dg*-*nk2.2* is only expressed in the posterior midgut. The light staining in the stomach of specimens labeled with *Dg*-*otx* and *Dg*-*dim* is an artefact, since precipitation was retained between the stomach content of the animals. *Dg*-*otx* and *Dg*-*pax6* are not shown in strict dorsoventral orientation, since the pharyngeal bulb is too extruded to balance the animals dorsoventrally at high magnification.

## Abbreviations

acom: anterior commissure
an: anus
br: brain
cb: ciliary band
cbg: ciliary band gland
cmvn: circumesophageal connective joining the medioventral nerve posteriorly
com: segmental commissure/commissural set
cvln: circumesophageal connective joining the ventrolateral nerve cords
drcc: dorsal root of the circumesophageal commissure
eso: esophagus
ey: eye
fog: foregut
fg: frontal gland
fpk: perikaryon with FMRFamide-like immunoreactivity(LIR)
lpmn: lateral paramedian nerve
mcom: median commissure
mo: mouth opening
mpmn: median paramedian nerve
mv: medioventral nerve
m1: segmental midbody ganglion
nc: nerve linking ciliary structure to the neuropil
no: nuchal organ
np: neuropil
pcb: prostomial ciliary band
pcc: prostomial compound cilia
pcom: posterior commissure
pg: prostomial ganglion
phb: pharyngeal bulb
ptr: prototroch
vnc: ventral nerve cord
pyg: pygidium
r1-r4: segmental rostral ganglion
se: surface ectoderm
scom: subrectal commissure
spk: perikaryon with serotonin-like immunoreactivity (LIR)
stnr: stomatogastric nerve ring
sto: stomach
tcom: terminal commissure
vcf: ventral ciliary field
vlnc: ventrolateral nerve cord
vrcc: ventral root of the circumesophageal connective
