# Supplementary material for: Molecular regionalization in the compact brain of the meiofaunal annelid Dinophilus gyrociliatus (Dinophilidae)
Source: EvoDevo. 2016 Aug 30;7(1):20. doi: 10.1186/s13227-016-0058-2 (PMC5006589; doi:10.1186/s13227-016-0058-2)
Supplement: Supplementary file 1 — 10.1186/s13227-016-0058-2 Sequences for gene orthology assignment. Amino acid sequences for Dinophilus gyrociliatus genes used in this study as well as related proteins from other animals, retrieved from NCBI (html://http://ncbi.nlm.nih.gov) and Joint Genome Institute (http://genome.jgi-psf.org/Capca1/Capca1.home.html for Capitella teleta; http://genome.jgi-psf.org/Lotgi1/Lotgi1.home.html for Lottia gigantea; http://genome.jgi-psf.org/Nemve1/Nemve1.home.html for Nematostella vectensis; http://genome.jgi-psf.org/Triad1/Triad1.home.html for Trichoplax adhaerens) including all information for the Additional files 3, 4, 5, 6, 7 and 8. http://genome.jgi-psf.org/Capca1/Capca1.home.html for Capitella teleta; for Lottia gigantea; for Nematostella vectensis; for Trichoplax adhaerens) including all information for the Additional files 3, 4, 5, 6, 7 and 8. [file 13227_2016_58_MOESM1_ESM.docx]

| **Gene** | **Animal** | **Length** | **GenBank Accession Number** | **Reference** |
| --- | --- | --- | --- | --- |
| **PAIRED CLASS GENES: OTP, OTX, GSC and HBN** | | | | |
| otp | *Dinophilus gyrociliatus* | 425(253)aa | KX555476 | this study |
| otx | *Dinophilus gyrociliatus* | 766(266)aa | KX555477 | this study |
| gsc | *Dinophilus gyrociliatus* | 223(184)aa | KX555474 | this study |
| hbn | *Dinophilus gyrociliatus* | 465(237)aa | KX555475 | this study |
| pax6 | *Dinophilus gyrociliatus* | 562(458)aa | KX555478 | this study |
| pitx | *Crepidula fornicata* | 369aa | ADI48168.1 | [1] |
| PREDICTED: otx1 | *Ciona intestinalis* | 284aa | XP_002119699.1 |  |
| TF: otp? | *Ciona intestinalis* | 492aa | NP_001072023.1 | [2] |
| otx | *Ciona intestinalis* | 425aa | NP_001027662.2 | [2] |
| EBX | *Capitella teleta* | 355aa | ABC58683.1 | [3] |
| gsx | *Capitella teleta* | 149aa | AAZ23124.1 | [3] |
| pax3/7 | *Capitella teleta* | 416aa | ABC68267.1 | [4] |
| xlox | *Capitella teleta* | 278aa | AAZ95509.1 | [3] |
| cdx | *Capitella teleta* | 338aa | AAZ95508.1 | [3] |
| ptx1, isoform A | *Drosophila melanogaster* | 509aa | NP_733410.2 | [5] |
| vsx1, isoform A | *Drosophila melanogaster* | 837aa | NP_572232.2 | [5] |
| vsx2, isoform A | *Drosophila melanogaster* | 640aa | NP_001033832.1 | [5] |
| aristaless | *Drosophila melanogaster* | 408aa | NP_722629.1 | [5] |
| dll, isoform A | *Drosophila melanogaster* | 327aa | NP_523857.1 | [5] |
| gsc, isoform C | *Drosophila melanogaster* | 415aa | NP_476949.2 | [5] |
| hbn | *Drosophila melanogaster* | 409aa | NP_788420.1 | [5] |
| otd | *Drosophila melanogaster* | 542aa | P22810.2 | [5] |
| otp | *Drosophila melanogaster* | 409aa | P56672.2 | [5] |
| repo | *Drosophila melanogaster* | 612aa | NP_477026.1 | [5] |
| rx | *Drosophila melanogaster* | 904aa | NP_726006.3 | [5] |
| otx | *Hydroides elegans* | 368aa | ABK76302.1 | [6] |
| arx | *Platynereis dumerilii* | 283aa | ADG26723.1 | [7] |
| paraHox, cdx | *Platynereis dumerilii* | 170aa | ACH87546.1 | [8] |
| paraHox, gsx | *Platynereis dumerilii* | 307aa | ACH87540.1 | [8] |
| pax6 | *Platynereis dumerilii* | 449aa | CAJ40659.1 | [9] |
| ParaHox, xlox | *Platynereis dumerilii* | 254aa | ACH87551.1 | [8] |
| Distalless, dlx-1 | *Platynereis dumerilii* | 367aa | CAJ38799.1 | [9] |
| Homeobox tf, gsc | *Platynereis dumerilii* | 279aa | CAC19336.1 | [10] |
| otp | *Platynereis dumerilii* | 267aa | ABR68849.1 | [11] |
| Homeobox transcription factor, otx | *Platynereis dumerilii* | 205aa | CAC19028.1 | [10] |
| rx | *Platynereis dumerilii* | 304aa | AAU20320.1 | [12] |
| gsc | *Patella vulgata* | 251aa | CAD45551.1 | [13] |
| otp | *Patella vulgata* | 254aa | AAM33145.1 | [14] |
| otx | *Patella vulgata* | 287aa | AAM33144.1 | [14] |
| Prop | *Saccoglossus kowalevskii* | 262aa | NP_001161635.1 |  |
| PREDICTED: arx | *Saccoglossus kowalevskii* | 379aa | XP_002731203.1 |  |
| otx | *Saccoglossus kowalevskii* | 307aa | NP_001158360.1 | [15] |
| otp | *Saccoglossus kowalevskii* | 322aa | NP_001158374.1 | [15] |
| rx1 | *Strongylocentrotus purpuratus* | 446aa | XP_781057.2 |  |
| PREDICTED: otp, isoform X2 | *Strongylocentrotus purpuratus* | 365aa | XP_784599.1 |  |
| otx, isoform alpha | *Strongylocentrotus purpuratus* | 371aa | NP_999753.2 | [16] |
| pax6 | *Terebratalia transversa* | 433aa | ADZ24784.1 | [17] |
| hbn | *Terebratalia transversa* | 323aa | AEZ03833.1 | [18] |
| otp | *Terebratalia transversa* | 302aa | AEZ03829.1 | [18] |
| otx | *Terebratalia transversa* | 270aa | ADZ24785.1 | [17] |
| **FOX-GENES** | | | | |
| foxG | *Dinophilus gyrociliatus* | 433(339)aa | KX555479 | this study |
| foxj1 | *Dinophilus gyrociliatus* | 837(430)aa |  | unpublished |
| foxQ2-1 | *Dinophilus gyrociliatus* | 329(299)aa |  | unpublished |
| foxD | *Dinophilus gyrociliatus* | 503(343)aa |  | unpublished |
| foxB | *Dinophilus gyrociliatus* | 464(241)aa |  | unpublished |
| foxN2/3 | *Dinophilus gyrociliatus* | 2069(1184)aa |  | unpublished |
| foxK | *Dinophilus gyrociliatus* | 780(444)aa |  | unpublished |
| foxJ2/3 | *Dinophilus gyrociliatus* | 691(441)aa |  | unpublished |
| foxC | *Dinophilus gyrociliatus* | 1558(433)aa |  | unpublished |
| foxN1/4 | *Dinophilus gyrociliatus* | 2069(300)aa |  | unpublished |
| foxL1 | *Dinophilus gyrociliatus* | 700(254)aa |  | unpublished |
| foxL2 | *Dinophilus gyrociliatus* | 331(249)aa |  | unpublished |
| foxQ2a | *Clytia hemisphaerica* | 387aa | ABG21224.1 | [19] |
| foxQ2b | *Clytia hemisphaerica* | 380aa | ABG21225.1 | [19] |
| foxA | *Capitella teleta* | 253aa | ABV25953.1 | [20] |
| foxB | *Capitella teleta* | 254aa | ELT97594.1 | [21] |
| foxAB | *Capitella teleta* | 118a | ELU18648.1 | [21] |
| foxC | *Capitella teleta* | 110aa | ELU02638.1 | [21] |
| foxD | *Capitella teleta* | 117aa | ELT88034.1 | [21] |
| foxF | *Capitella teleta* | 105aa | ELT88831.1 | [21] |
| foxG | *Capitella teleta* | 157aa | ELT94106.1 | [21] |
| foxGa | *Capitella teleta* | 326aa | ELT94108.1 | [21] |
| foxI | *Capitella teleta* | 253aa | ELU09837.1 | [21] |
| foxJ1 | *Capitella teleta* | 528aa | ELT89986.1 | [21] |
| foxJ2/3 | *Capitella teleta* | 144aa | ELU06126.1 | [21] |
| foxK | *Capitella teleta* | 389aa | ELU13569.1 | [21] |
| foxL1 | *Capitella teleta* | 102aa | ELU02639.1 | [21] |
| foxL2 | *Capitella teleta* | 92aa | ELU18532.1 | [21] |
| foxM | *Capitella teleta* | 108aa | ELU13054.1 | [21] |
| foxN1/4 | *Capitella teleta* | 89aa | ELT98166.1 | [21] |
| foxN2/3 | *Capitella teleta* | 228aa | ELU16338.1 | [21] |
| foxO | *Capitella teleta* | 121aa | ELU03603.1 | [21] |
| foxP | *Capitella teleta* | 547aa | ELT95700.1 | [21] |
| foxQ1 | *Capitella teleta* | 293aa | ELT93183.1 | [21] |
| foxQ2a | *Capitella teleta* | 104aa | ELU17556.1 | [21] |
| foxQ2b | *Capitella teleta* | 249aa | ELU00476.1 | [21] |
| 110212 | *Nematostella vectensis* | 116aa | XP_001631592.1 | [22] |
| 118122 | *Nematostella vectensis* | 101aa | XP_001629014.1 | [22] |
| 120142 | *Nematostella vectensis* | 157aa | XP_001628369.1 | [22] |
| 121754 | *Nematostella vectensis* | 130aa | XP_001627842.1 | [22] |
| 123903 | *Nematostella vectensis* | 285aa | XP_001627122.1 | [22] |
| 125256 | *Nematostella vectensis* | 95aa | XP_001626709.1 | [22] |
| 132285 | *Nematostella vectensis* | 430aa |  |  |
| 138488 | *Nematostella vectensis* | 95aa | XP_001623177.1 | [22] |
| 150900 | *Nematostella vectensis* | 63aa | XP_001619589.1 | [22] |
| 161006 | *Nematostella vectensis* | 213aa | XP_001638960.1 | [22] |
| 165261 | *Nematostella vectensis* | 286aa | XP_001634555.1 | [22] |
| 165603 | *Nematostella vectensis* | 277aa |  |  |
| 18324 | *Nematostella vectensis* | 100aa | XP_001630413.1 | [22] |
| 187332 | *Nematostella vectensis* | 262aa | XP_001631625.1 | [22] |
| 19405 | *Nematostella vectensis* | 182aa | XP_001631844.1 | [22] |
| 201028 | *Nematostella vectensis* | 285aa |  |  |
| 213966 | *Nematostella vectensis* | 280aa | XP_001627679.1 | [22] |
| 228732 | *Nematostella vectensis* | 2503aa | XP_001626353.1 | [22] |
| 38679 | *Nematostella vectensis* | 87aa | XP_001639907.1 | [22] |
| 39596 | *Nematostella vectensis* | 97aa | XP_001637106.1 | [22] |
| 39632 | *Nematostella vectensis* | 101aa | XP_001637167.1 | [22] |
| 5001 | *Nematostella vectensis* | 86aa | XP_001624096.1 | [22] |
| 58039 | *Nematostella vectensis* | 98aa | XP_001634717.1 | [22] |
| 65438 | *Nematostella vectensis* | 94aa | XP_001636939.1 | [22] |
| 67043 | *Nematostella vectensis* | 104aa | XP_001639875.1 | [22] |
| 67209 | *Nematostella vectensis* | 95aa | XP_001641147.1 | [22] |
| 88569 | *Nematostella vectensis* | 101aa | XP_001638891.1 | [22] |
| 99317 | *Nematostella vectensis* | 101aa | XP_001637168.1 | [22] |
| 96685 | *Nematostella vectensis* | 92aa | XP_001636048.1 | [22] |
| foxA | *Lottia gigantea* | 437aa | XP_009063695.1 | [21] |
| foxAB | *Lottia gigantea* | 105aa | XP_009047947.1 | [21] |
| foxB | *Lottia gigantea* | 110aa | XP_009048711.1 | [21] |
| foxC | *Lottia gigantea* | 106aa | XP_009054250.1 | [21] |
| foxD | *Lottia gigantea* | 107aa | XP_009045977.1 | [21] |
| foxF | *Lottia gigantea* | 151aa | XP_009054244.1 | [21] |
| foxG | *Lottia gigantea* | 105aa | XP_009046406.1 | [21] |
| foxH | *Lottia gigantea* | 72aa | XP_009066217.1 | [21] |
| foxJ1 | *Lottia gigantea* | 98aa | XP_009059936.1 | [21] |
| foxJ1 | *Lottia gigantea* | 85aa | XP_009063460.1 | [21] |
| foxJ2/3 | *Lottia gigantea* | 151aa | XP_009058107.1 | [21] |
| foxK | *Lottia gigantea* |  |  |  |
| foxL1 | *Lottia gigantea* | 144aa | XP_009054249.1 | [21] |
| foxL2 | *Lottia gigantea* | 90aa | XP_009066965.1 | [21] |
| foxN2/3 | *Lottia gigantea* | 208aa | XP_009047023.1 | [21] |
| foxP | *Lottia gigantea* | 254aa | XP_009057794.1 | [21] |
| foxQ2 | *Lottia gigantea* | 99a | XP_009045446.1 | [21] |
| foxA1 (hepatocyte nuclear factor 3-beta isoform b) | *Mus musculus* | 468aa | NP_032285.2 | [23] |
| foxA2 (hepatocyte nuclear factor 3-beta isoform b) | *Mus musculus* | 459aa | NP_034576.2 | [24] |
| foxB1 | *Mus musculus* | 325aa | NP_071773.2 | [25] |
| foxB2 | *Mus musculus* | 428aa | NP_032049.1 | [26] |
| foxC1 | *Mus musculus* | 553aa | NP_032618.2 | [27] |
| foxC2 | *Mus musculus* | 494aa | NP_038547.2 | [27] |
| foxD2 | *Mus musculus* | 492aa | NP_032619.1 | [28] |
| foxD3 | *Mus musculus* | 469aa | NP_034555.3 | [29] |
| foxD4 | *Mus musculus* | 444aa | NP_032048.1 | [30] |
| foxE1 | *Mus musculus* | 371aa | NP_899121.1 | [31] |
| foxE3 | *Mus musculus* | 288aa | NP_056573.1 | [32] |
| foxF1 | *Mus musculus* | 378aa | NP_034556.2 | [33] |
| foxF2 | *Mus musculus* | 446aa | NP_034355.2 | [33] |
| foxG1 | *Mus musculus* | 481aa | NP_032267.1 | [34] |
| foxH1 | *Mus musculus* | 401aa | NP_032015.1 | [35] |
| foxI1 | *Mus musculus* | 372aa | NP_076396.3 | [36] |
| foxI2 | *Mus musculus* | 329aa | NP_899016.1 | [37] |
| foxJ1 | *Mus musculus* | 421aa | NP_032266.3 | [38] |
| foxJ2 | *Mus musculus* | 565aa | NP_068699.1 | [39] |
| foxJ3 isoform 1 | *Mus musculus* | 623aa | NP_766287.1 | [40] |
| foxK1 | *Mus musculus* | 719aa | NP_951031.2 | [41] |
| foxK2 | *Mus musculus* | 651aa | NP_001074401.2 | [39] |
| foxM1 | *Mus musculus* | 757aa | NP_032047.4 | [42] |
| foxN1 isoform 1 | *Mus musculus* | 648aa | NP_032264.1 | [43] |
| foxN2 | *Mus musculus* | 429aa | NP_851305.2 | [44] |
| foxN4 | *Mus musculus* | 521aa | NP_683737.2 | [45] |
| foxO1 | *Mus musculus* | 652aa | NP_062713.2 | [46] |
| foxO3 | *Mus musculus* | 672aa | NP_062714.1 | [47] |
| foxO6 | *Mus musculus* | 559aa | NP_918949.1 | [48] |
| foxP1 isoform 1 | *Mus musculus* | 705aa | NP_444432.1 | [49] |
| foxP2 | *Mus musculus* | 714aa | NP_997600.1 | [42] |
| foxP3 | *Mus musculus* | 429aa | NP_473380.1 | [50] |
| foxP4 isoform 3 | *Mus musculus* | 672aa | NP_083043.2 | [51] |
| foxQ1 | *Mus musculus* | 400aa | NP_032265.3 | [52] |
| foxS1 | *Mus musculus* | 329aa | NP_034356.1 | [53] |
| foxG | *Platynereis dumerilii* | 328aa | ADG26725.1 | [7] |
| foxQ2 | *Ptychodera flava* | 247aa | ADZ61650.1 | [54] |
| PREDICTED foxR1 | *Rattus norvegicus* | 384aa | XP_243815.4 | [55] |
| PREDICTED foxR2 | *Rattus norvegicus* | 302aa | XP_228808.3 | [55] |
| foxI | *Strongylocentrotus purpuratus* | 316aa | ABB89485.1 | [55] |
| foxL1 | *Strongylocentrotus purpuratus* | 431aa | ABB89488.1 | [55] |
| foxA | *Strongylocentrotus purpuratus* | 440aa | ABE68834.1 | [55] |
| foxAB | *Strongylocentrotus purpuratus* | 166aa | ABB89474.1 | [55] |
| foxB | *Strongylocentrotus purpuratus* | 360aa | NP_999797.1 | [55] |
| foxC | *Strongylocentrotus purpuratus* | 336aa | ABB89478.1 | [55] |
| foxD | *Strongylocentrotus purpuratus* | 367aa | ABB89476.1 | [55] |
| foxF | *Strongylocentrotus purpuratus* | 201aa | ABB89479.1 | [55] |
| foxG | *Strongylocentrotus purpuratus* | 185aa | ABB89477.1 | [55] |
| foxJ1 | *Strongylocentrotus purpuratus* | 468aa | ABB89480.1 | [55] |
| foxK | *Stronglocentrotus purpuratus* | 606aa | ABB89486.1 | [55] |
| foxL2 | *Strongylocentrotus purpuratus* | 372aa | ABB89483.1 | [55] |
| foxM | *Strongylocentrotus purpuratus* | 599aa | ABB89490.1 | [55] |
| foxN1/4 transcript variant 1 | *Strongylocentrotus purpuratus* | 532aa | ABB89491.1 | [55] |
| foxN2/3 | *Strongylocentrotus purpuratus* | 197aa | ABB89482.1 | [55] |
| foxO | *Strongylocentrotus purpuratus* | 476aa | ABB89484.1 | [55] |
| foxP | *Strongylocentrotus purpuratus* | 582aa | ABB89487.1 | [55] |
| foxQ1 | *Strongylocentrotus purpuratus* | 408aa | ABB89489.1 | [55] |
| foxQ2 | *Strongylocentrotus purpuratus* | 329aa | ABB89473.1 | [55] |
| foxG | *Terebratalia transversa* | 418aa | AEZ03828.1 | [18] |
| foxQ2 | *Terebratalia transversa* | 257aa | AEZ03835.1 | [18] |
| **SIX-CLASS GENES** | | | | |
| six3/6 | *Dinophilus gyrociliatus* | 482(398)aa | KX555480 | this study |
| 180297 | *Capitella teleta* | 314aa | ELU17224.1 | [21] |
| 180301 | *Capitella teleta* | 242aa | ELU17225.1 | [21] |
| 180303 | *Capitella teleta* | 346aa | ELU17231.1 | [21] |
| 226834 | *Capitella teleta* | 371aa | ELU17226.1 | [21] |
| 227938 | *Capitella teleta* | 506aa | ELT94068.1 | [21] |
| optix | *Drosophila melanogaster* | 487aa | NP_524695.2 | [5] |
| sine oculis | *Drosophila melanogaster* | 416aa | NP_476733.1 | [5] |
| six4, isoform A | *Drosophila melanogaster* | 392aa | NP_649256.1 | [5] |
| 115798 | *Lottia gigantea* | 222aa | XP_009052787.1 | [56] |
| 129577 | *Lottia gigantea* | 205aa | XP_009063131.1 | [56] |
| 179424 | *Lottia gigantea* | 191aa | XP_009063129.1 | [56] |
| six1 | *Mus musculus* | 284aa | NP_033215.2 | [57] |
| six2 | *Mus musculus* | 296aa | NP_035510.1 | [58] |
| six3 | *Mus musculus* | 333aa | NP_035511.2 | [59] |
| sSix4 | *Mus musculus* | 775aa | NP_035512.1 | [57] |
| sSix5 | *Mus musculus* | 719aa | NP_035513.1 | [39] |
| six6 | *Mus musculus* | 246aa | NP_035514.1 | [60] |
| 126214 | *Nematostella vectensis* | 185aa | XP_001626434.1 | [22] |
| 130873 | *Nematostella vectensis* | 192aa | XP_001625159.1 | [22] |
| 138693 | *Nematostella vectensis* | 175aa | XP_001623134.1 | [22] |
| six2 | *Platynereis dumerilii* | 307aa | CAC86663.1 | [61] |
| six3 | *Platynereis dumerilii* | 366aa | CAR66435.1 | [62] |
| six1 | *Saccoglossus kowalevskii* | 296aa | NP_001277017.1 | [63] |
| six3 | *Saccoglossus kowalevskii* | 252aa | NP_001158378.1 | [15] |
| PREDICTED: six4-like | *Saccoglossus kowalevskii* | 304aa | XP_002735606.1 |  |
| six1 | *Strongylocentrotus purpuratus* | 336aa | NP_001268684.1 | [64] |
| PREDICTED: six6-like, six3? | *Strongylocentrotus purpuratus* | 324aa | XP_781696.1 |  |
| PREDICTED: six4 | *Strongylocentrotus purpuratus* | 565aa | XP_781616.2 |  |
| sSix1/2 | *Terebratalia transversa* | 199aa | AJV21321.1 | [65] |
| six3/6 | *Terebratalia transversa* | 348aa | AEZ03831.1 | [18] |
| **DIMMED** | | | | |
| dimmed | *Dinophilus gyrociliatus* | 1212(161)aa | KX555473 | this study |
| dimmed, isoform A | *Drosophila melanogaster* | 390aa | AAF53991 | [66] |
| dimmed, isoform B | *Drosophila melanogaster* | 390aa | AGB93209.1 | [66] |
| dimmed, | *Leptinotarsa decemlineata* | 176aa | AKG92779.1 | [67] |
| dimmed transcription factor | *Platynereis dumerilii* | 205aa | AFV92894.1 | [68] |
| Hypothetical protein partial mRNA | *Lottia gigantea* | 327bp | XM_009067111.1 | [56] |
| PREDICTED: neurogenic differentiation factor 4-like, ... | *Crassostrea gigas* | 2009bp | XM_011430741.1 |  |
| Basic helix-loop-helix protein mist 1, putative | Pediculus humanus corporis | 300bp | XM_002432560.1 | [69] |
| dlx | *Nematostella vectensis* | 60aa | ABG67787.1 | [70] |
| dlx | *Paracentrotus lividus* | 419aa | ADW95343.1 | [71] |
| dlx | *Convolutriba longifissura* | 536aa | ACG70806.1 | [72] |
| dlx | *Saccoglossus kowalevskii* | 274aa | AAP79300.1 | [73] |
| **NK-HOMEOBOX GENES** | | | | |
| nk2.1 | *Dinophilus gyrociliatus* | 1360(210)aa | KX555481 | this study |
| nk2.2 | *Dinophilus gyrociliatus* | 347(290)aa | KX555482 | this study |
| lbx | *Capitella teleta* | 367aa | ACI26672.1 | [74] |
| nk-like2.2b | *Capitella teleta* | 221aa | ACH89433.1 | [74] |
| nk-like-1a | *Capitella teleta* | 148aa | ACH70609.1 | [74] |
| nk-like-1b | *Capitella teleta* | 165aa | ACI26669.1 | [74] |
| nk-like-2.1a | *Capitella teleta* | 263aa | ACH89430.1 | [74] |
| nk-like-2.2a | *Capitella teleta* | 256aa | ACH89432.1 | [74] |
| nk-like-2.1b | *Capitella teleta* | 428aa | ACH89431.1 | [74] |
| nk-like-3 | *Capitella teleta* | 190aa | ACI26670.1 | [74] |
| nk-like-4a | *Capitella teleta* | 237aa | ACH89434.1 | [74] |
| nk-like-4b | *Capitella teleta* | 297aa | ACH89435.1 | [74] |
| nk-like-5 | *Capitella teleta* | 402aa | ACH89437.1 | [74] |
| nk-like-5b | *Capitella teleta* | 207aa | ACH88440.1 | [74] |
| nk-like-6 | *Capitella teleta* | 146aa | ACI26668.1 | [74] |
| nk-like_7 | *Capitella teleta* | 267aa | ACI26671.1 | [74] |
| tlx | *Capitella teleta* | 271aa | ACH89436.1 | [74] |
| bap | *Drososphila melanogaster* | 382aa | NP_732637.1 | [5] |
| c15 | *Drososphila melanogaster* | 339aa | NP_476873.2 | [5] |
| dll | *Drososphila melanogaster* | 322aa | NP_726486.1 | [5] |
| drop | *Drososphila melanogaster* | 515aa | NP_477324.1 | [5] |
| h6 | *Drososphila melanogaster* | 592aa | NP_732244.3 | [5] |
| lbe, ladybird early | *Drososphila melanogaster* | 479aa | NP_524435.2 | [5] |
| lbl, ladybird late, isoform A | *Drososphila melanogaster* | 372aa | NP_524434.2 | [5] |
| scro, scarecrow, isoform A | *Drososphila melanogaster* | 468aa | NP_001015473.1 | [5] |
| slouch, isoform A | *Drososphila melanogaster* | 659aa | NP_476657.1 | [5] |
| tinman | *Drososphila melanogaster* | 416aa | NP_524433.1 | [5] |
| vnd (ventral nervous system defective), isoform B | *Drososphila melanogaster* | 577aa | NP_001036253.1 | [5] |
| dlx-1 | *Mus musculus* | 255aa | NP_034183.1 | [75] |
| dlx-2 | *Mus musculus* | 332aa | NP_034184.1 | [76] |
| dlx-3 | *Mus musculus* | 287aa | NP_034185.1 | [77] |
| dlx-4 | *Mus musculus* | 240aa | NP_031893.3 | [28] |
| dlx-5, isoform 1 | *Mus musculus* | 289aa | NP_034186.2 | [78] |
| dlx-6 | *Mus musculus* | 297aa | NP_034187.1 | [78] |
| hmx1 | *Mus musculus* | 332aa | NP_034575.1 | [79] |
| hmx2 | *Mus musculus* | 273aa | NP_666110.1 | [80] |
| hmx3 | *Mus musculus* | 356aa | NP_032283.3 | [80] |
| lbx1 | *Mus musculus* | 282aa | NP_034821.2 | [81] |
| lbx2 | *Mus musculus* | 195aa | NP_034822.1 | [82] |
| msx-1 | *Mus musculus* | 303aa | NP_034965.2 | [83] |
| msx-2 | *Mus musculus* | 267aa | NP_038629.2 | [84] |
| msx-3 | *Mus musculus* | 204aa | NP_034966.1 | [85] |
| nk2.5 | *Mus musculus* | 318aa | NP_032726.1 | [86] |
| nkx-3.2 | *Mus musculus* | 333aa | NP_031550.2 | [87] |
| nkx-3.1 | *Mus musculus* | 237aa | NP_035051.1 | [88] |
| nkx-6.1 | *Mus musculus* | 365aa | NP_659204.1 | [89] |
| nkx-6.2 | *Mus musculus* | 277aa | NP_899071.2 | [90] |
| nkx-6.3 | *Mus musculus* | 262aa | NP_083278.1 | [91] |
| nkx-1.1 | *Mus musculus* | 440aa | NP_035450.1 | [92] |
| nkx-1.2 | *Mus musculus* | 305aa | NP_033149.1 | [93] |
| nkx-2.1 | *Mus musculus* | 372aa | NP_033411.3 | [94] |
| nkx-2.2 | *Mus musculus* | 273aa | NP_035049.1 | [95] |
| nkx-2.3 | *Mus musculus* | 362aa | NP_032725.1 | [96] |
| nkx-2.4 | *Mus musculus* | 354aa | NP_075993.1 | [97] |
| nkx-2.6 | *Mus musculus* | 289aa | NP_035050.2 | [98] |
| nkx-2.8 | *Mus musculus* | 235aa | NP_032727.2 | [99] |
| tlx1 | *Mus musculus* | 333aa | NP_068701.1 | [100] |
| tlx2 | *Mus musculus* | 284aa | NP_033418.1 | [28] |
| tlx3 | *Mus musculus* | 291aa | NP_064300.2 | [84] |
| dlx-1 | *Platynereis dumerilii* | 367aa | CAJ38799.1 | [9] |
| lbx | *Platynereis dumerilii* | 497aa | ABQ10642.2 | [101] |
| msx | *Platynereis dumerilii* | 389aa | CAJ38810.1 | [9] |
| nk1 | *Platynereis dumerilii* | 333aa | CAJ38797.1 | [9] |
| nk2.1 | *Platynereis dumerilii* | 362aa | CAJ38809.1 | [9] |
| nk2.2 | *Platynereis dumerilii* | 200aa | ABO93209.1 | [102] |
| nk3 | *Platynereis dumerilii* | 387aa | ABQ10641.1 | [101] |
| nk4 | *Platynereis dumerilii* | 374aa | ABQ10640.1 | [101] |
| nk5 | *Platynereis dumerilii* | 171aa | ABQ10644.1 | [101] |
| tlx | *Platynereis dumerilii* | 325aa | ABQ10643.1 | [101] |
| nk2.1 | *Terebratalia transversa* | 323aa | AEZ03832.1 | [18] |
| **SYNAPTOTAGMIN** | | | | |
| syt | *Dinophilus gyrociliatus* | 816(414)aa | KX555483 | this study |
| dblc2 | *Capitella teleta* | 411aa | ELT93877.1 | [56] |
| esyt2 | *Capitella teleta* | 825aa | ELU09670.1 | [56] |
| rabphilin | *Capitella teleta* | 672aa | ELT93620.1 | [56] |
| syt12 | *Capitella teleta* | 454aa | ELT99116.1 | [56] |
| syt15 | *Capitella teleta* | 307aa | ELU10846.1 | [56] |
| syt16 | *Capitella teleta* | 424aa | ELU17232.1 | [56] |
| syt17 | *Capitella teleta* | 483aa | ELU18173.1 | [56] |
| syt1var1 | *Capitella teleta* | 437aa | ELT99110.1 | [56] |
| syt1var2 | *Capitella teleta* | 437aa | ELT99111.1 | [56] |
| syt4 | *Capitella teleta* | 416aa | ELT89176.1 | [56] |
| syt44 | *Capitella teleta* | 414aa | ELT95572.1 | [56] |
| syt7 | *Capitella teleta* | 336aa | ELT91365.1 | [56] |
| syt9 | *Capitella teleta* | 468aa | ELU04478.1 | [56] |
| sytalpha | *Capitella teleta* | 323aa | ELT91518.1 | [56] |
| dblc2 | *Lottia gigantea* | 405aa | XP_009066759.1 | [56] |
| esyt2var1 | *Lottia gigantea* | 807aa | XP_009052922.1 | [56] |
| esyt2var2 | *Lottia gigantea* | 798aa | XP_009052923.1 | [56] |
| syt12 | *Lottia gigantea* | 411aa | XP_009064408.1 | [56] |
| syt15a | *Lottia gigantea* | 537aa | XP_009056107.1 | [56] |
| syt15b | *Lottia gigantea* | 353aa | XP_009060412.1 | [56] |
| syt16 | *Lottia gigantea* | 501aa | XP_009055193.1 | [56] |
| syt 17 | *Lottia gigantea* | 437aa | XP_009061942.1 | [56] |
| syt18 | *Lottia gigantea* | 394aa | XP_009061817.1 | [56] |
| syt1var1 | *Lottia gigantea* | 424aa | XP_009065902.1 | [56] |
| syt1var2 | *Lottia gigantea* | 424aa | XP_009065903.1 | [56] |
| syt21 | *Lottia gigantea* | 354aa | XP_009052921.1 | [56] |
| syt4 | *Lottia gigantea* | 423aa | XP_009048108.1 | [56] |
| syt47 | *Lottia gigantea* | 430aa | XP_009058888.1 | [56] |
| syt7 | *Lottia gigantea* | 446aa | XP_009065399.1 | [56] |
| syt9 | *Lottia gigantea* | 452aa | XP_009048259.1 | [56] |
| sytalpha | *Lottia gigantea* | 486aa | XP_009063446.1 | [56] |
| dblc2 | *Mus musculus* | 405aa | NP_034199.1 | [102] |
| rph3A | *Mus musculus* | 681aa | NP_035416.1 | [103] |
| syt1, isoform 1 | *Mus musculus* | 421aa | NP_033332.1 | [104] |
| syt11 | *Mus musculus* | 430aa | NP_061274.2 | [105] |
| syt10 | *Mus musculus* | 523aa | NP_061273.1 | [106] |
| syt12 | *Mus musculus* | 421aa | NP_598925.1 | [107] |
| syt13 | *Mus musculus* | 426aa | NP_109650.1 | [108] |
| syt14, isoform 2 | *Mus musculus* | 555aa | NP_853524.1 | [109] |
| syt15, isoform a | *Mus musculus* | 418aa | NP_852682.1 | [98] |
| syt16 | *Mus musculus* | 549aa | NP_766392.2 | [98] |
| syt17 | *Mus musculus* | 470aa | NP_619590.1 | [110] |
| syt2 | *Mus musculus* | 422aa | NP_033333.2 | [111] |
| syt3 | *Mus musculus* | 587aa | NP_057872.3 | [98] |
| syt4 | *Mus musculus* | 425aa | NP_033334.2 | [112\| |
| syt5 | *Mus musculus* | 386aa | NP_058604.1 | [28] |
| syt6, isoform 1 | *Mus musculus* | 511aa | NP_061270.2 | [98] |
| syt7, alpha isoform | *Mus musculus* | 403aa | NP_061271.1 | [113] |
| syt8, isoform a | *Mus musculus* | 395aa | NP_061272.2 | [98] |
| syt9 | *Mus musculus* | 491aa | NP_068689.2 | [98] |
| syt1 | *Terebratalia transversa* | 341aa | AEZ03834.1 | [18] |

**References:**

1. Henry, J. J., Perry, K. J., Fukui, L. & Alvi, N. Differential localization of mRNAs during early development in the mollusc, *Crepidula fornicata*. *Integr. Comp. Biol.* **50,** 720–733 (2010).

2. Satou, Y., Kawashima, T., Shoguchi, E., Nakayama, A. & Satoh, N. An integrated database of the ascidian, *Ciona intestinalis*: Towards functional genomics. *Zool. Sci.* **22,** 837–843 (2005).

3. Fröbius, A. C. & Seaver, E. C. *ParaHox* gene expression in the polychaete annelid *Capitella* sp. I. *Dev. Genes Evol.* **216,** 81–88 (2006).

4. Seaver, E. C., Yamaguchi, E., Richards, G. S. & Meyer, N. P. Expression of the pair-rule gene homologs *runt*, *Pax3/7*, *even-skipped-1* and *even-skipped-2* during larval and juvenile development of the polychaete annelid *Capitella teleta* does not support a role in segmentation. *Evodevo* **3,** (2012).

5. Hoskins, R. A. *et al.* Sequence finishing and mapping of *Drosophila melanogaster* heterochromatin. *Science* **316,** 1625–1628 (2007).

6. Arenas-Mena, C. & Wong, K. S.-Y. *HeOtx* expression in an indirectly developing polychaete correlates with gastrulation by invagination. *Dev. Genes Evol.* **217,** 373–384 (2007).

7. Tomer, R., Denes, A. S., Tessmar-Raible, K. & Arendt, D. Profiling by Image Registration reveals common origin of annelid mushroom bodies and vertebrate pallium. *Cell* **142,** 800–809 (2010).

8. Hui, J. H. L. *et al.* Features of the ancestral bilaterian inferred from *Platynereis dumerilii* ParaHox genes. *BMC Biol* **7,** 43–13 (2009).

9. Raible, F. *et al.* Vertebrate-type intron-rich genes in the marine annelid *Platynereis dumerilii*. *Science* **310,** 1325–1326 (2005).

10. Arendt, D., Technau, U. & Wittbrodt, J. Evolution of the bilaterian larval foregut. *Nature* **409,** 81–85 (2001).

11. Tessmar-Raible, K. *et al.* Conserved sensory-neurosecretory cell types in annelid and fish forebrain: Insights into hypothalamus evolution. *Cell* **129,** 1389–1400 (2007).

12. Arendt, D., Tessmar-Raible, K., Snyman, H., Dorresteijn, A. W. & Wittbrodt, J. Ciliary photoreceptors with a vertebrate-type Opsin in an invertebrate brain. *Science* **306,** 869–871 (2004).

13. Lartillot, N., Le Gouar, M. & Adoutte, A. Expression patterns of *fork head* and *goosecoid* homologues in the mollusc *Patella vulgata* supports the ancestry of the anterior mesendoderm across Bilateria. *Dev. Genes Evol.* **212,** 551–561 (2002).

14. Nederbragt, A., Welscher, te, P., van den Driesche, S., van Loon, A. X. & Dictus, W. Novel and conserved roles for *orthodenticle/otx* and *orthopedia/otp* orthologs in the gastropod mollusc *Patella vulgata*. *Dev. Genes Evol.* **212,** 330–337 (2002).

15. Lowe, C. J. *et al.* Anteroposterior patterning in hemichordates and the origins of the chordate nervous system. *Cell* **113,** 853–865 (2003).

16. Tu, Q., Cameron, R. A., Worley, K. C., Gibbs, R. A. & Davidson, E. H. Gene structure in the sea urchin *Strongylocentrotus purpuratus* based on transcriptome analysis. *Genome Res.* **22,** 2079–2087 (2012).

17. Passamaneck, Y. J., Furchheim, N., Hejnol, A., Martindale, M. Q. & Lueter, C. Ciliary photoreceptors in the cerebral eyes of a protostome larva. *Evodevo* **2,** (2011).

18. Santagata, S., Resh, C., Hejnol, A., Martindale, M. Q. & Passamaneck, Y. J. Development of the larval anterior neurogenic domains of *Terebratalia transversa* (Brachiopoda) provides insights into the diversification of larval apical organs and the spiralian nervous system. *Evodevo* **3,** 1–21 (2012).

19. Chevalier, S., Martin, A., Leclere, L., Amiel, A. & Houliston, E. Polarised expression of *FoxB* and *FoxQ2* genes during development of the hydrozoan *Clytia hemisphaerica*. *Dev. Genes Evol.* **216,** 709–720 (2006).

20. Boyle, M. J. & Seaver, E. C. Developmental expression of *foxA* and *gata* genes during gut formation in the polychaete annelid, *Capitella* sp I. *Evolution & Development* **10,** 89–105 (2008).

21. Grigoriev, I. V. *et al.* The genome portal of the Department of Energy Joint Genoe Institute. *Nucleic Acid Reearch* **40,** D26–32 (2012).

22. Putnam, N. H. *et al.* Sea anemone genome reveals ancestral eumetazoan gene repertoire and genomic organization. *Science* **317,** 86–94 (2007).

23. Liu, Y. *et al.* FoxA1 directs the lineage and immunosuppressive properties of a novel regulatory T cell population in EAE and MS. *Nat. Med.* **20,** 272–282 (2014).

24. Yi, S.-H. *et al.* Foxa2 acts as a co-activator potentiating expression of the Nurr1-induced DA phenotype via epigenetic regulation. *Development* **141,** 761–772 (2014).

25. Uchibe, K., Shimizu, H., Yokoyama, S., Kuboki, T. & Asahara, H. Identification of novel transcription-regulating genes expressed during murine molar development. *Dev. Dyn.* **241,** 1217–1226 (2012).

26. Hammock, E. A. D. *et al.* Homologs of genes expressed in *Caenorhabditis elegans* GABAergic neurons are also found in the developing mouse forebrain. *Neural Dev* **5,** (2010).

27. Kuracha, M. R., Siefker, E., Licht, J. D. & Govindarajan, V. Spry1 and Spry2 are necessary for eyelid closure. *Dev. Biol.* **383,** 227–238 (2013).

28. Diez-Roux, G. *et al.* A high-resolution anatomical atlas of the transcriptome in the mouse embryo. *PLoS Biol* **9,** (2011).

29. Pasek, R. C. & Gannon, M. Advancements and challenges in generating accurate animal models of gestational diabetes mellitus. *American Journal of Physiology-Endocrinology and Metabolism* **305,** E1327–E1338 (2013).

30. Pazin, D. E., Gamer, L. W., Cox, K. A. & Rosen, V. Molecular profiling of synovial joints: Use of microarray analysis to identify factors that direct the development of the knee and elbow. *Dev. Dyn.* **241,** 1816–1826 (2012).

31. Hick, A.-C. *et al.* Reciprocal epithelial:endothelial paracrine interactions during thyroid development govern follicular organization and C-cells differentiation. *Dev. Biol.* **381,** 227–240 (2013).

32. Carbe, C. *et al.* An allelic series at the paired box gene 6 (*Pax6*) locus reveals the functional specificity of *Pax* genes. *J. Biol. Chem.* **288,** 12130–12141 (2013).

33. Hoggatt, A. M. *et al.* The transcription factor *Foxf1* binds to serum response factor and myocardin to regulate gene transcription in visceral smooth muscle cells. *J. Biol. Chem.* **288,** 28477–28487 (2013).

34. Watson, L. A. *et al.* Dual effect of CTCF loss on neuroprogenitor differentiation and survival. *J. Neurosci.* **34,** 2860–2870 (2014).

35. Redshaw, N. *et al.* TGF-beta/Smad2/3 signaling directly regulates several miRNAs in mouse ES cells and early embryos. *PLoS ONE* **8,** (2013).

36. Yu, J. *et al.* Identification of molecular compartments and genetic circuitry in the developing mammalian kidney. *Development* **139,** 1863–1873 (2012).

37. Shibata M, Blauvelt KE, Liem KF, Garcia-Garcia MJ. TRIM28 is required by the mouse KRAB domain protein ZFP568 to control convergent extension and morphogenesis of extra-embryonic tissues. Development. 2011;138:5333–43.

38. Tan FE, Vladar EK, Ma L, Fuentealba LC, Hoh R, Espinoza FH, et al. *Myb* promotes centriole amplification and later steps of the multiciliogenesis program. Development. 2013;140:4277–86.

39. Chung YC, Tsai YJ, Shiu T-Y, Sun Y-Y, Wang P-F, Chen C-L. Screening large numbers of expression patterns of transcription factors in late stages of the mouse thymus. Gene Expression Patterns. 2011;11:84–92.

40. Yamamoto H, Morino K, Nishio Y, Ugi S, Yoshizaki T, Kashiwagi A, et al. MicroRNA-494 regulates mitochondrial biogenesis in skeletal muscle through mitochondrial transcription factor A and Forkhead box j3. American Journal of Physiology-Endocrinology and Metabolism. 2012;303:E1419–27.

41. Sel S, Münzenberg C, Nass N, Kalinski T, Datan M, Auffarth GU, et al. The transcription factor Foxk1 is expressed in developing and adult mouse neuroretina. Gene Expression Patterns. 2013;13:280–6.

42. Wu X, Gu X, Han X, Du A, Jiang Y, Zhang X, et al. A novel function for *Foxm1* in interkinetic nuclear migration in the developing telencephalon and anxiety-related dehavior. J. Neurosci. Society for Neuroscience; 2014;34:1510–22.

43. Acute ablation of DP thymocytes induces up-regulation of IL-22 and Foxn1 in TECs. 2014;150:101–8.

44. Wu J, Bao J, Kim M, Yuan S, Tang C, Zheng H, et al. Two miRNA clusters, miR-34b/c and miR-449, are essential for normal brain development, motile ciliogenesis, and spermatogenesis. Proc. Natl. Acad. Sci. USA. National Acad Sciences; 2014;111:E2851–7.

45. Misra K, Luo H, Li S, Matise M, Xiang M. Asymmetric activation of Dll4-Notch signaling by Foxn4 and proneural factors activates BMP/TGF signaling to specify V2b interneurons in the spinal cord. Development. 2013;141:187–98.

46. Wimmer RJ, Liu Y, Schachter TN, Stonko DP, Peercy BE, Schneider MF. Mathematical modeling reveals modulation of both nuclear influx and efflux of Foxo1 by the IGF-I/PI3K/Akt pathway in skeletal muscle fibers. American Journal of Physiology-Cell Physiology. 2014;306:C570–84.

47. Pietras EM, Lakshminarasimhan R, Techner J-M, Fong S, Flach J, Binnewies M, et al. Re-entry into quiescence protects hematopoietic stem cells from the killing effect of chronic exposure to type I interferons. J. Exp. Med. Rockefeller Univ Press; 2014;211:245–62.

48. Kim DH, Zhang T, Lee S, Calabuig-Navarro V, Yamauchi J, Piccirillo A, et al. FoxO6 integrates insulin signaling with MTP for regulating VLDL production in the liver. Endocrinology. Endocrine Society Chevy Chase, MD; 2014;155:1255–67.

49. Svoboda DS, Paquin A, Park DS, Slack RS. Pocket proteins pRb and p107 are required for cortical lamination independent of apoptosis. Dev. Biol. 2013;384:101–13.

50. Mu J, Tai X, Iyer SS, Weissman JD, Singer A, Singer DS. Regulation of MHC Class I expression by Foxp3 and its effect on regulatory T Cell function. J. Immunol. American Association of Immunologists; 2014;192:2892–903.

51. Otero JJ, Kalaszczynska I, Michowski W, Wong M, Gygli PE, Gokozan HN, et al. Cerebellar cortical lamination and foliation require cyclin A2. Dev. Biol. 2014;385:328–39.

52. Wu B, Pratt CH, Potter CS, Silva KA, Kennedy VE, Sandberg JP. R164C mutation in FOXQ1 H3 domain affects formation of the hair medulla. Journal of Investigative Dermatology. 2013;133:S82–2.

53. Pihlajoki M, Gretzinger E, Cochran R, Kyronlahti A, Schrade A, Hiller T, et al. Conditional mutagenesis of Gata6 in SF1-positive cells causes gonadal-like differentiation in the adrenal cortex of mice. Endocrinology. Endocrine Society Chevy Chase, MD; 2013;154:1754–67.

54. Röttinger E, Martindale MQ. Ventralization of an indirect developing hemichordate by NiCl2 suggests a conserved mechanism of dorso-ventral (D/V) patterning in Ambulacraria (hemichordates and echinoderms). Dev. Biol. 2011;354:173–90.

55. Tu Q, Brown CT, Davidson EH, Oliveri P. Sea urchin Forkhead gene family: Phylogeny and embryonic expression. Dev. Biol. 2006;300:49–62.

56. Simakov O, Marletaz F, Cho S-J, Edsinger-Gonzales E, Havlak P, Hellsten U, et al. Insights into bilaterian evolution from three spiralian genomes. Nature. Nature Publishing Group; 2013;493:526–31.

57. Daou N, Lecolle S, Lefebvre S, Gaspera BD, Charbonnier F, Chanoine C, et al. A new role for the calcineurin/NFAT pathway in neonatal myosin heavy chain expression via the NFATc2/MyoD complex during mouse myogenesis. Development. 2013;140:4914–25.

58. Xu J, Liu H, Park J-S, Lan Y, Jiang R. *Osr1* acts downstream of and interacts synergistically with *Six2* to maintain nephron progenitor cells during kidney organogenesis. Development. 2014;141:1442–52.

59. Li L, Liu C, Biechele S, Zhu Q, Song L, Lanner F, et al. Location of transient ectodermal progenitor potential in mouse development. Development. 2013;140:4533–43.

60. Trowe MO, Zhao L, Weiss AC, Christoffels V, Epstein DJ, Kispert A. Inhibition of Sox2-dependent activation of *Shh* in the ventral diencephalon by Tbx3 is required for formation of the neurohypophysis. Development. 2013;140:2299–309.

61. Arendt D, Tessmar K, Medeiros de Campos-Baptista M-I, Dorresteijn AW, Wittbrodt J. Development of pigment-cup eyes in the polychaete *Platynereis dumerilii* and evolutionary conservation of larval eyes in Bilateria. Development. 2002;129:1143–54.

62. Steinmetz PRH, Urbach R, Posnien N, Eriksson J, Kostyuchenko RP, Brena C, et al. *Six3* demarcates the anterior-most developing brain region in bilaterian animals. Evodevo. BioMed Central Ltd; 2010;1:1–9.

63. Gillis JA, Fritzenwanker JH, Lowe CJ. A stem-deuterostome origin of the vertebrate pharyngeal transcriptional network. Proceedings: Biological Sciences. The Royal Society; 2012;279:237–46.

64. Tu Q, Cameron RA, Worley KC, Gibbs RA, Davidson EH. Gene structure in the sea urchin *Strongylocentrotus purpuratus* based on transcriptome analysis. Genome Res. Cold Spring Harbor Lab; 2012;22:2079–87.

65. Passamaneck YJ, Hejnol A, Martindale MQ. Mesodermal gene expression during the embryonic and larval development of the articulate brachiopod *Terebratalia transversa*. Evodevo. 10 ed. 2015;6.

66. Adams MD, Celniker SE, Holt RA, Evans CA, Gocayne JD, Amanatides PG, et al. The Genome Sequence of Drosophila melanogaster. Science. American Association for the Advancement of Science; 2000;287:2185–95.

67. Meng, Q. and Fu, K. (2014): Identification of bHLH genes in Colorado potato beatle, Leptinotarsa decemlineata (direct submission)

68. Conzelmann M, Williams EA, Tunaru S, Randel N, Shahidi R, Asadulina A, et al. Conserved MIP receptor–ligand pair regulates *Platynereis* larval settlement. PNAS. 2013;110:8224–9.

69. Kirkness, E. et al. 2007: Annotation of Pediculus humanus corporis strain USDA (unpublished)

70. Chourrout D, Delsuc F, Chourrout P, Edvardsen RB, Rentzsch F, Renfer E, et al. Minimal ProtoHox cluster inferred from bilaterian and cnidarian Hox complements. Nature. Nature Publishing Group; 2006;442:684–7.

71. Saudemont A, Haillot E, Mekpoh F, Bessodes N, Quirin M, Lapraz F, et al. Ancestral regulatory circuits governing ectoderm patterning downstream of Nodal and BMP2/4 revealed by gene regulatory network analysis in an echinoderm. Martindale M, editor. PLoS Genet. Public Library of Science; 2010;6.

72. Hejnol A, Martindale MQ. Acoel development indicates the independent evolution of the bilaterian mouth and anus. Nature. Nature Publishing Group; 2008;456:382–U45.

73. Lowe CJ, Terasaki M, Wu M, Freeman RM, Runft L, Kwan K, et al. Dorsoventral patterning in hemichordates: Insights into early chordate evolution. De Robertis EM, editor. PLoS Biol. 2006;4:1603–19.

74. Veatch,O.J. and Seaver,E.C.(2008): Genomic organization and expression of NK-like gene families in Capitella sp. I. Unpublished

75. Song H, Lee B, Pyun D, Guimera J, Son Y, Yoon J, et al. *Ascl1* and *Helt* act combinatorially to specify thalamic neuronal identity by repressing *Dlxs* activation. Dev. Biol. Elsevier; 2015;398:280–91.

76. Li X, Venugopalan SR, Cao H, Pinho FO, Paine ML, Snead ML, et al. A model for the molecular underpinnings of tooth defects in Axenfeld-Rieger syndrome. Hum. Mol. Genet. Oxford University Press; 2014;23:194–208.

77. Yang G, Yuan G, Li X, Liu P, Chen Z, Fan M. BMP-2 induction of Dlx3 expression is mediated by p38/Smad5 signaling pathway in osteoblastic MC3T3-E1 cells. Journal of Cellular Physiology. 2014;229:943–54.

78. Berghoff EG, Clark MF, Chen S, Cajigas I, Leib DE, Kohtz JD. *Evf2* (*Dlx6as*) lncRNA regulates ultraconserved enhancer methylation and the differential transcriptional control of adjacent genes. Development. Oxford University Press for The Company of Biologists Limited; 2013;140:4407–16.

79. Boulling A, Wicht L, Schorderet DF. Identification of *HMX1* target genes: A predictive promoter model approach. Mol. Vis. Molecular Vision; 2013;19:1779–94.

80. Romand R, Krezel W, Beraneck M, Cammas L, Fraulob V, Messaddeq N, et al. Retinoic acid deficiency impairs the vestibular function. J. Neurosci. Society for Neuroscience; 2013;33:5856–81. Paixao S, Balijepalli A, Serradj N, Niu J, Luo W, Martin JH, et al. EphrinB3/EphA4-mediated guidance of sscending and descending spinal tracts. Neuron. 2013;80:1407–20.

82. Gerber SD, Amann R, Wyder S, Trueb B. Comparison of the gene expression profiles from formal and *Fgfrl1* deficient mouse kidneys reveals downstream targets of *Fgfrl1* signaling. Rishi A, editor. PLoS ONE. Public Library of Science; 2012;7.

83. Forni PE, Bharti K, Flannery EM, Shimogori T, Wray S. The indirect role of fibroblast growth factor-8 in defining neurogenic niches of the olfactory/GnRH systems. J. Neurosci. Society for Neuroscience; 2013;33:19620–34.

84. Marklund U, Alekseenko Z, Andersson E, Falcii S, Westgren M, Perlmann T, et al. Detailed expression analysis of regulatory genes in the early developing human neural tube. Stem Cells and Development. Mary Ann Liebert, Inc. 140 Huguenot Street, 3rd Floor New Rochelle, NY 10801 USA; 2013;23:5–15.

85. Singh N, Gupta M, Trivedi CM, Singh MK, Li L, Epstein JA. Murine craniofacial development requires Hdac3-mediated repression of *Msx* gene expression. Dev. Biol. 2013;377:333–44.

86. Wetzel-Strong SE, Li M, Nishikimi T, Caron KM. Epicardial-derived adrenomedullin drives cardiac hyperplasia during embryogenesis. Circulation Research. Lippincott Williams & Wilkins; 2013;113:–A052.

87. Huang H, Cotton JL, Wang Y, Rajurkar M, Zhu LJ, Lewis BC, et al. Specific requirement of *Gli* transcription factors in *hedgehog*-mediated intestinal development. J. Biol. Chem. American Society for Biochemistry and Molecular Biology; 2013;288:17589–96.

88. Kruithof-de Julio M, Shibata M, Desai N, Reynon M, Halili MV, Hu Y-P, et al. Canonical *Wnt* signaling regulates *Nkx3.1* expression and luminal epithelial differentiation during prostate organogenesis. Dev. Dyn. 2013;242:1160–71.

89. Gaber ZB, Butler SJ, Novitch BG. PLZF regulates fibroblast growth factor responsiveness and maintenance of neural progenitors. Polleux F, editor. PLoS Biol. Public Library of Science; 2013;11.

90. Wang B, Long JE, Flandin P, Pla R, Waclaw RR, Campbell K, et al. Loss of Gsx1 and Gsx2 function rescues distinct phenotypes in Dlx1/2 mutants. J. Comp. Neurol. Wiley Subscription Services, Inc., A Wiley Company; 2013;521:1561–84.

91. Du A, McCracken KW, Walp ER, Terry NA, Klein TJ, Han A, et al. *Arx* is required for normal enteroendocrine cell development in mice and humans. Dev. Biol. Elsevier Inc; 2012;365:175–88.

92. Simon R, Britsch S, Bergemann A. Ablation of *Sax2* gene expression prevents diet-induced obesity. FEBS J. Blackwell Publishing Ltd; 2011;278:371–82.

93. Buniello A, Hardisty-Hughes RE, Pass JC, Bober E, Smith RJ, Steel KP. Headbobber: A combined morphogenetic and cochleosaccular mouse model to study 10qter deletions in human deafness. Zhang X, editor. PLoS ONE. Public Library of Science; 2013;8.

94. Hines EA, Jones M-KN, Verheyden JM, Harvey JF, Sun X. Establishment of smooth muscle and cartilage juxtaposition in the developing mouse upper airways. Proc. Natl. Acad. Sci. USA. National Acad Sciences; 2013;110:19444–9.

95. Zhu Q, Zhao X, Zheng K, Li H, Huang H, Zhang Z, et al. Genetic evidence that *Nkx2.2* and *Pdgfra* are major determinants of the timing of oligodendrocyte differentiation in the developing CNS. Development. 2014;141:548–55.

96. Norden J, Greulich F, Rudat C, Taketo MM, Kispert A. Wnt/beta-Catenin signaling maintains the mesenchymal precursor pool for murine sinus horn formation. Circulation Research. 2011;109:e42–e50.

97. Pereira JD, Sansom SN, Smith J, Dobenecker M-W, Tarakhovsky A, Livesey FJ, et al. Ezh2, the histone methyltransferase of PRC2, regulates the balance between self-renewal and differentiation in the cerebral cortex. Proceedings of the National Academy of Sciences of the United States of America. National Academy of Sciences; 2010;107:15957–62.

98. Skarnes WC, Rosen B, West AP, Koutsourakis M, Bushell W, Iyer V, et al. A conditional knockout resource for the genome-wide study of mouse gene function. Nature. Nature Publishing Group; 2011;474:337–61.

99. Herriges JC, Yi L, Hines EA, Harvey JF, Xu G, Gray PA, et al. Genome-scale study of transcription factor expression in the branching mouse lung. Dev. Dyn. Wiley‐Liss, Inc; 2012;241:1432–53.

100. Swinehart IT, Schlientz AJ, Quintanilla CA, Mortlock DP, Wellik DM. *Hox11* genes are required for regional patterning and integration of muscle, tendon and bone. Development. Oxford University Press for The Company of Biologists Limited; 2013;140:4574–82.

101. Saudemont A, Dray N, Hudry B, Le Gouar M, Vervoort M, Balavoine G. Complementary striped expression patterns of *NK* homeobox genes during segment formation in the annelid *Platynereis*. Dev. Biol. 2008;317:430–43.

102. Denes AS, Jekely G, Steinmetz PRH, Raible F, Snyman H, Prud'homme B, et al. Molecular architecture of annelid nerve cord supports common origin of nervous system centralization in bilateria. Cell. 2007;129:277–88.

103. Brozzi F, Lajus S, Diraison F, Rajatileka S, Hayward K, Regazzi R, et al. MyRIP interaction with MyoVa on secretory granules is controlled by the cAMP-PKA pathway. Mol. Biol. Cell. American Society for Cell Biology; 2012;23:4444–55.

104. Wierda KDB, Sorensen JB. Innervation by a GABAergic neuron depresses spontaneous release in glutamatergic neurons and unveils the clamping phenotype of Synaptotagmin-1. J. Neurosci. Society for Neuroscience; 2014;34:2100–10.

105. Duque GA, Fukuda M, Descoteaux A. Synaptotagmin XI regulates phagocytosis and cytokine secretion in macrophages. J. Immunol. American Association of Immunologists; 2013;190:1737–45.

106. Cao P, Yang X, Suedhof TC. Complexin activates exocytosis of distinct secretory vesicles controlled by different synaptotagmins. J. Neurosci. Society for Neuroscience; 2013;33:1714–27.

107. Kaeser-Woo YJ, Younts TJ, Yang X, Zhou P, Wu D, Castillo PE, et al. Synaptotagmin-12 phosphorylation by cAMP-dependent protein kinase Is essential for hippocampal mossy fiber LTP. J. Neurosci. Society for Neuroscience; 2013;33:9769–80.

108. Han S, Hong S, Lee D, Lee M-H, Choi J-S, Koh MJ, et al. Altered expression of synaptotagmin 13 mRNA in adult mouse brain after contextual fear conditioning. Biochemical and Biophysical Research Communications. Elsevier Inc; 2012;425:880–5.

109. Fukuda M. Molecular cloning, expression, and characterization of a novel class of synaptotagmin (Syt XIV) conserved from *Drosophila* to humans. J. Biochem. Oxford University Press; 2003;133:641–9.

110. Huang X, Dubuc AM, Hashizume R, Berg J, He Y, Wang J, et al. Voltage-gated potassium channel EAG2 controls mitotic entry and tumor growth in medulloblastoma via regulating cell volume dynamics. Genes Dev. Cold Spring Harbor Lab; 2012;26:1780–96.

111. Gundersen CB, Umbach JA. Synaptotagmins 1 and 2 as mediators of rapid exocytosis at nerve terminals: The dyad hypothesis. J. Theor. Biol. 2013;332:149–60.

112. Dean C, Liu H, Staudt T, Stahlberg MA, Vingill S, Bueckers J, et al. Distinct subsets of *Syt-IV/BDNF* vesicles are sorted to axons versus dendrites and recruited to synapses by activity. J. Neurosci. Society for Neuroscience; 2012;32:5398–413.

113. Liu H, Bai H, Hui E, Yang L, Evans CS, Wang Z, et al. Synaptotagmin 7 functions as a Ca2+-sensor for synaptic vesicle replenishment. Elife. 2014;3.
