# Supplementary material for: Molecular regionalization in the compact brain of the meiofaunal annelid Dinophilus gyrociliatus (Dinophilidae)
Source: EvoDevo. 2016 Aug 30;7(1):20. doi: 10.1186/s13227-016-0058-2 (PMC5006589; doi:10.1186/s13227-016-0058-2)
Supplement: Supplementary file 2 — 10.1186/s13227-016-0058-2 Primer sequences used for the respective genes in Dinophilus gyrociliatus. Matching primer pairs were designed using MacVector (MACVECTOR, INC., Cambridge, UK) and the “Sequencing Primers/Probes”-tool. Pairs were chosen based on their length (20–25 bp), G-C-content (45–55 %, manually checked for not more than 3 C or G in a row) and similar working temperature. [file 13227_2016_58_MOESM2_ESM.docx]

| **Gene** | **Sense** | **Antisense** |
| --- | --- | --- |
| *Dg-dim* | 5'- ATCGTTTGTCGTTCCGTG -3' | 5'- TGAGAGGCAATAGGTGTGC -3' |
| *Dg-foxg* | 5'- TAGAGATGACGAGAGACGGCAG -3' | 5'- CGACGATTCAATGCTGAC -3' |
| *Dg-gsc* | Race primer 1 5'- TTCTCGCCTTTTCTTCTCCTGTTCTTCACG -3'  Race primer 2 5'- TGTTCGGTGTCTTCGTTTCCTCTTGTGACC -3' |  |
| *Dg-hbn* | 5'- CAAAGCGAACTAAACGACAG -3' | 5'- GCACTCTTTGTCACACCAAG -3' |
| *Dg-nk2.1* | 5'- CCCTCCCTCCTCGGTTTTAC -3' | 5'- AGCGCCTATACAACTCTGGA -3' |
| *Dg-nk2.2* | 5'- TAGAGATGACGAGAGACGGCAG -3' | 5'- TAGAGATGACGAGAGACGGCAG -3' |
| *Dg-otp* | 5'- CTAAACGCTGATTGGTGTG -3' | 5'- AAATCCACTGTGACCCATC -3' |
| *Dg-otx* | 5'- ATTGAGGCCAGCGAAATGAC -3' | 5'- GACAACACCGCTCTTTTCCA -3' |
| *Dg-pax6* | 5'- CAAGCCTGAAGAACCAAAG -3’ | 5'- TGATGTATGGGCGAATAGAC -3’ |
| *Dg-six3/6* | 5'- TTACCCCGCCTCAAATAGCT -3' | 5'- CGGCATTGGTATTGTAGCGT -3' |
| *Dg-syt* | 5'- GAGTTAGCAAAGGAGCCAG -3' | 5'- ACCGTAATGATAAGCGTCAC -3' |
