# Supplementary figures and images for: Molecular regionalization in the compact brain of the meiofaunal annelid Dinophilus gyrociliatus (Dinophilidae)
Source: EvoDevo. 2016 Aug 30;7(1):20. doi: 10.1186/s13227-016-0058-2 (PMC5006589; doi:10.1186/s13227-016-0058-2)

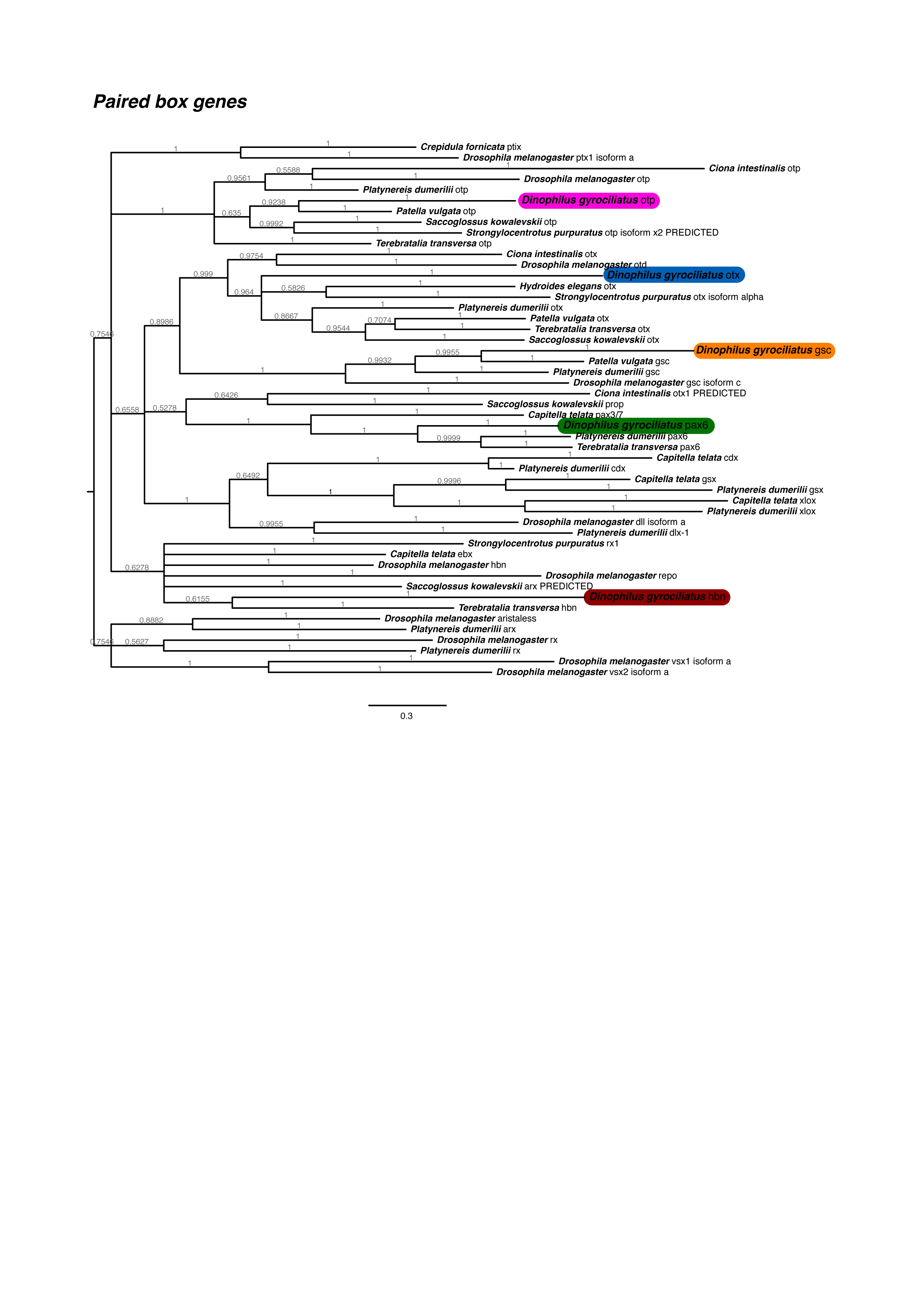

Supplement: Supplementary file 3 — 10.1186/s13227-016-0058-2 Phylogenetic analysis of Dg-syt. Phylogenetic tree of the membrane-trafficking protein SYNAPTOTAGMIN-1 with emphasis on Dg-syt (which is highlighted in the tree), supporting its orthology assignment. Protein alignments were made using MUSCLE [67] and Bayesian phylogenetic analysis was performed using MrBayes [68], with settings according to [22]. Each analysis was run for 30,000,000 generations sampled every 1000 generations in four runs. A consensus tree and posterior probabilities for each branch were calculated prior to visualization of the tree with FigTree and edition in Adobe Illustrator 2015CC. All sequences used are listed in Additional file 1. [file 13227_2016_58_MOESM3_ESM.tif]

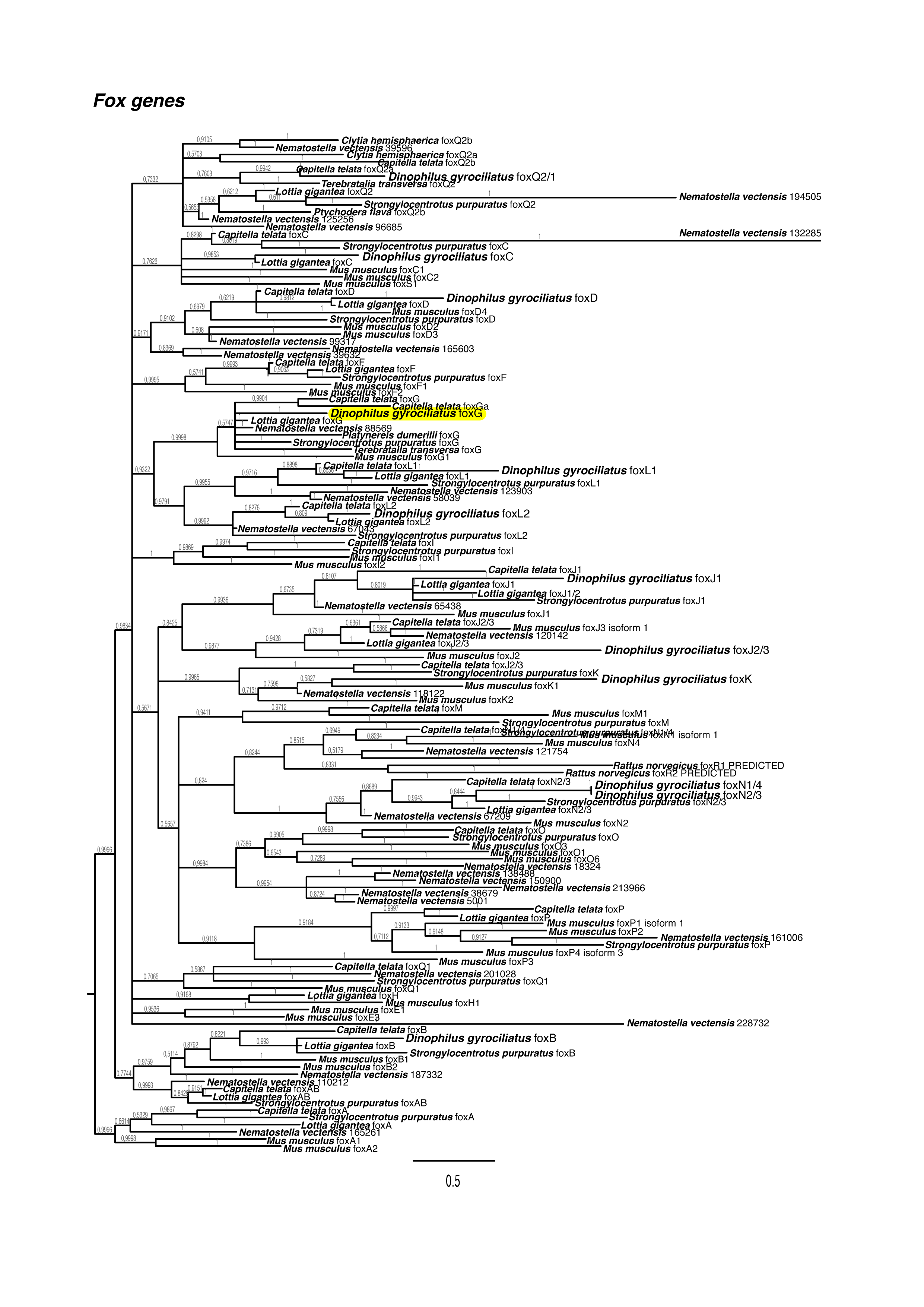

Supplement: Supplementary file 4 — 10.1186/s13227-016-0058-2 Phylogenetic analysis of PRD class genes Dg-gsc, Dg-hbn, Dg-otp, Dg-otx and Dg-pax6. Phylogenetic tree of the paired box genes with emphasis on the genes used in this study (which are highlighted in the tree), supporting their orthology assignments. Protein alignments were made using MUSCLE [67] and Bayesian phylogenetic analysis was performed using MrBayes [68], with settings according to [22]. Each analysis was run for 30,000,000 generations sampled every 1000 generations in four runs. A consensus tree and posterior probabilities for each branch were calculated prior to visualization of the tree with FigTree and edition in Adobe Illustrator 2015CC. All sequences used are listed in Additional file 2. [file 13227_2016_58_MOESM4_ESM.tif]

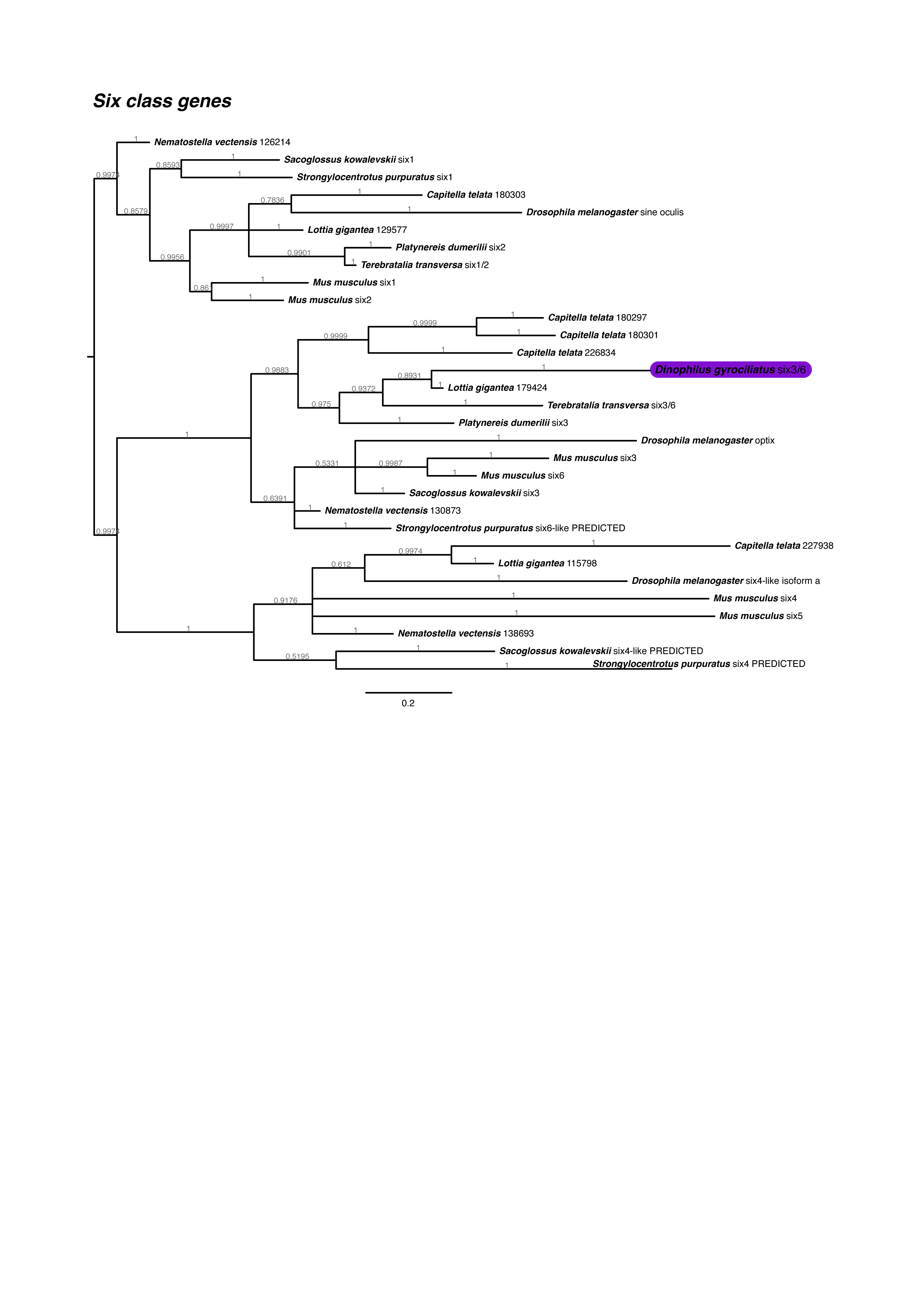

Supplement: Supplementary file 5 — 10.1186/s13227-016-0058-2 Phylogenetic analysis of Dg-foxG. Phylogenetic tree of the forkhead box genes with emphasis on Dg-foxG (which is highlighted in the tree), supporting its orthology assignments. Protein alignments were made using MUSCLE [67] and Bayesian phylogenetic analysis was performed using MrBayes [68], with settings according to [22]. Each analysis was run for 30,000,000 generations sampled every 1000 generations in four runs. A consensus tree and posterior probabilities for each branch were calculated prior to visualization of the tree with FigTree and edition in Adobe Illustrator 2015CC. All sequences used are listed in Additional file 1. [file 13227_2016_58_MOESM5_ESM.tif]

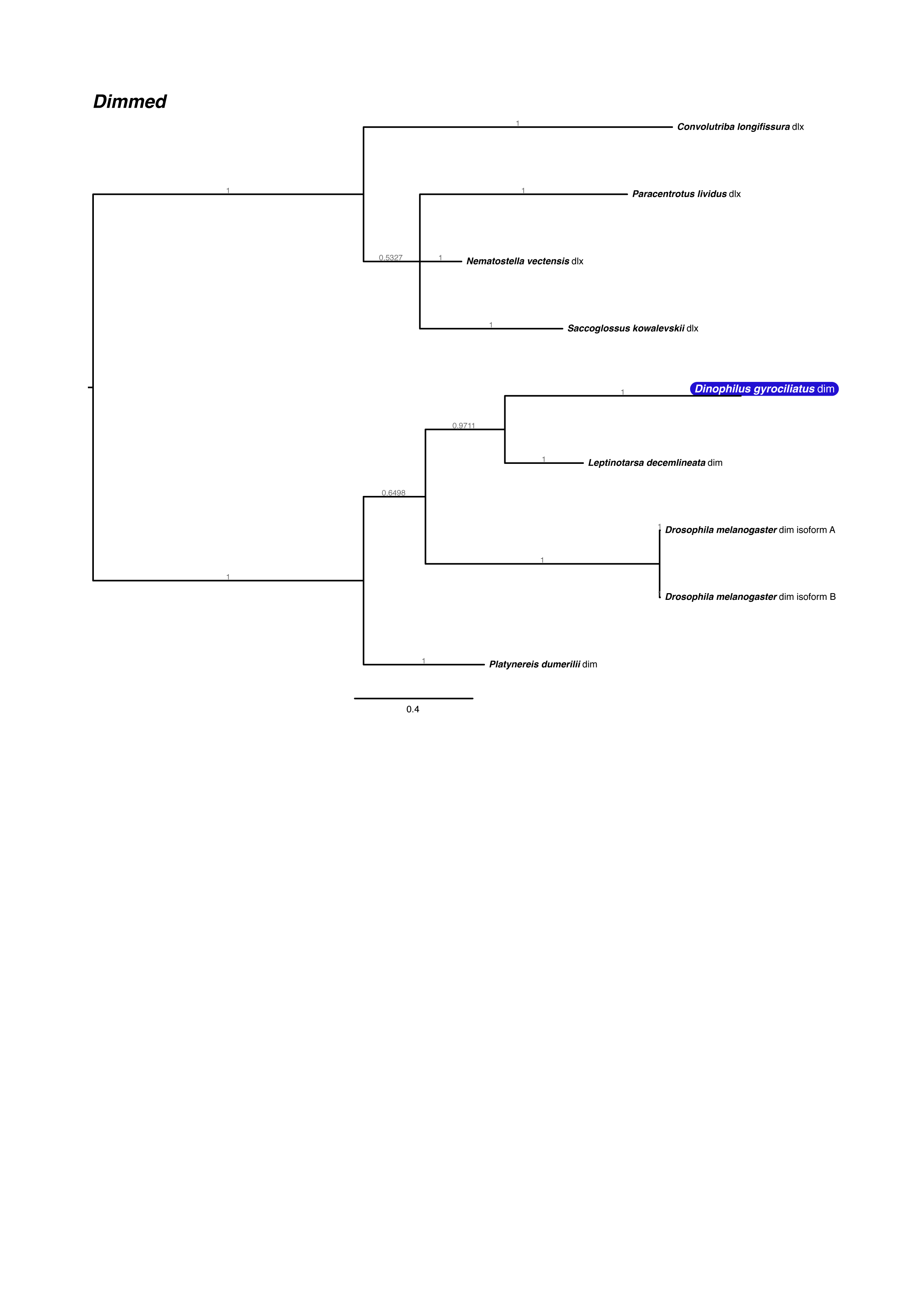

Supplement: Supplementary file 6 — 10.1186/s13227-016-0058-2 Phylogenetic analysis of Dg-six3/6. Phylogenetic tree of the six class genes with emphasis on Dg-six3/6 (which is highlighted in the tree), supporting its orthology assignments. Protein alignments were made using MUSCLE [67] and Bayesian phylogenetic analysis was performed using MrBayes [68], with settings according to [22]. Each analysis was run for 30,000,000 generations sampled every 1000 generations in four runs. A consensus tree and posterior probabilities for each branch were calculated prior to visualization of the tree with FigTree and edition in Adobe Illustrator 2015CC. All sequences used are listed in Additional file 1. [file 13227_2016_58_MOESM6_ESM.tif]

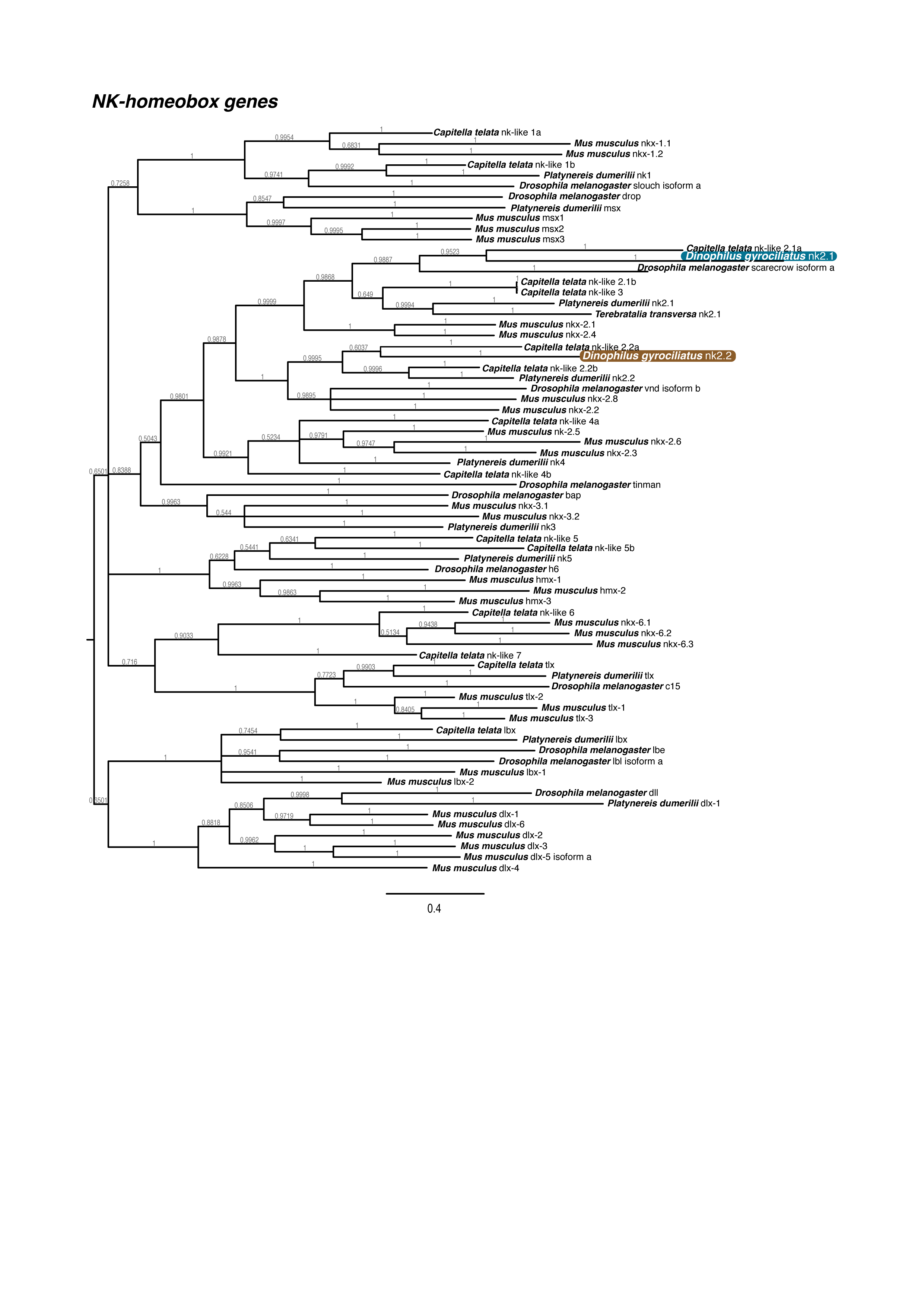

Supplement: Supplementary file 7 — 10.1186/s13227-016-0058-2 Phylogenetic analysis of Dg-dim. Phylogenetic tree of the dimmed genes with emphasis on Dg-dim (which is highlighted in the tree), supporting its orthology assignments. Protein alignments were made using MUSCLE [67] and Bayesian phylogenetic analysis was performed using MrBayes [68], with settings according to [22]. Each analysis was run for 30,000,000 generations sampled every 1000 generations in four runs. A consensus tree and posterior probabilities for each branch were calculated prior to visualization of the tree with FigTree and edition in Adobe Illustrator 2015CC. All sequences used are listed in Additional file 1. [file 13227_2016_58_MOESM7_ESM.tif]

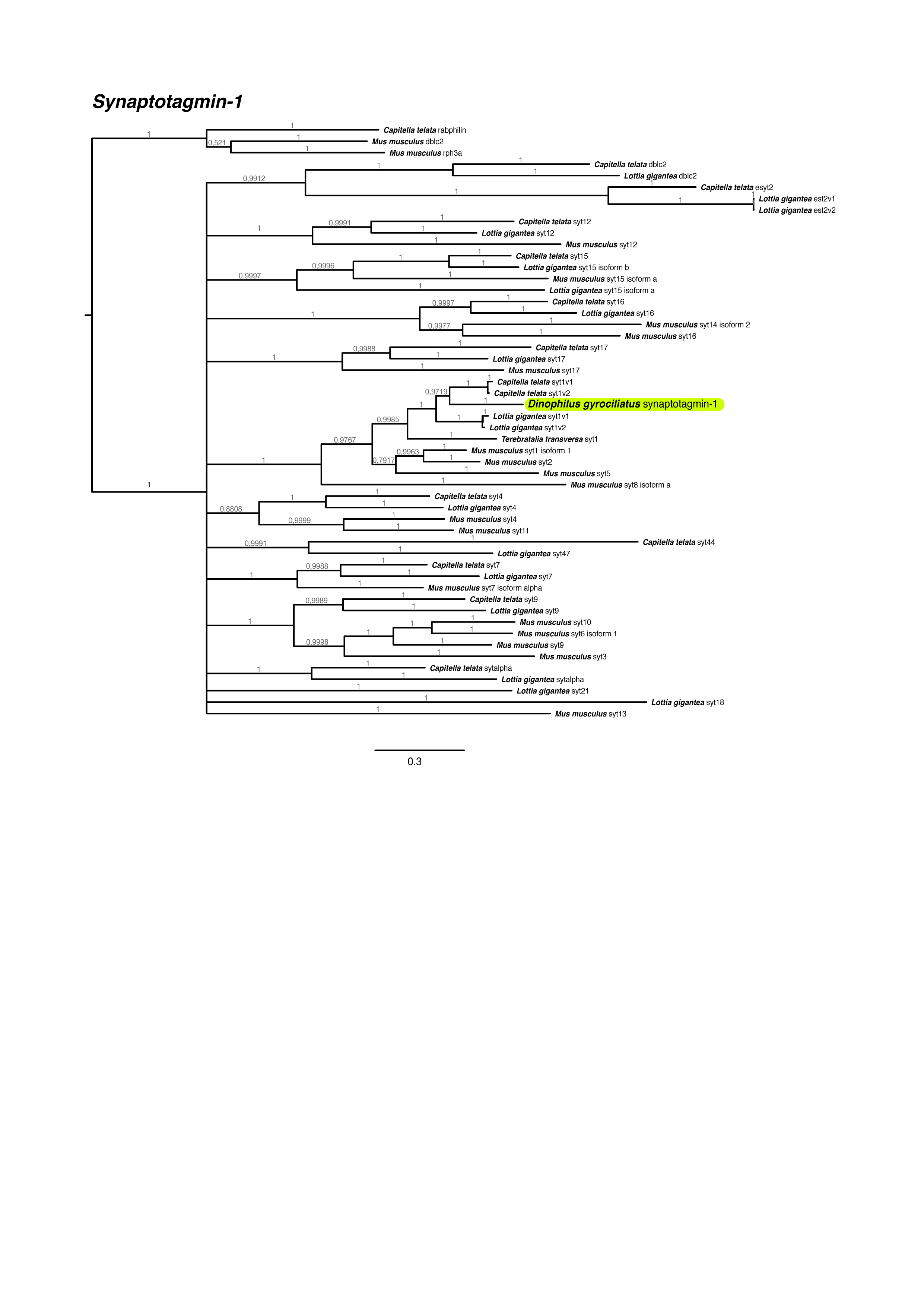

Supplement: Supplementary file 8 — 10.1186/s13227-016-0058-2 Phylogenetic analysis of Dg-nk2.1 and Dg-nk2.2. Phylogenetic tree of the NK-homeobox genes with emphasis on the genes used in this study (which are highlighted in the tree), supporting their orthology assignments. Protein alignments were made using MUSCLE [67] and Bayesian phylogenetic analysis was performed using MrBayes [68], with settings according to [22]. Each analysis was run for 30,000,000 generations sampled every 1000 generations in four runs. A consensus tree and posterior probabilities for each branch were calculated prior to visualization of the tree with FigTree and edition in Adobe Illustrator 2015CC. All sequences used are listed in Additional file 1. [file 13227_2016_58_MOESM8_ESM.tif]

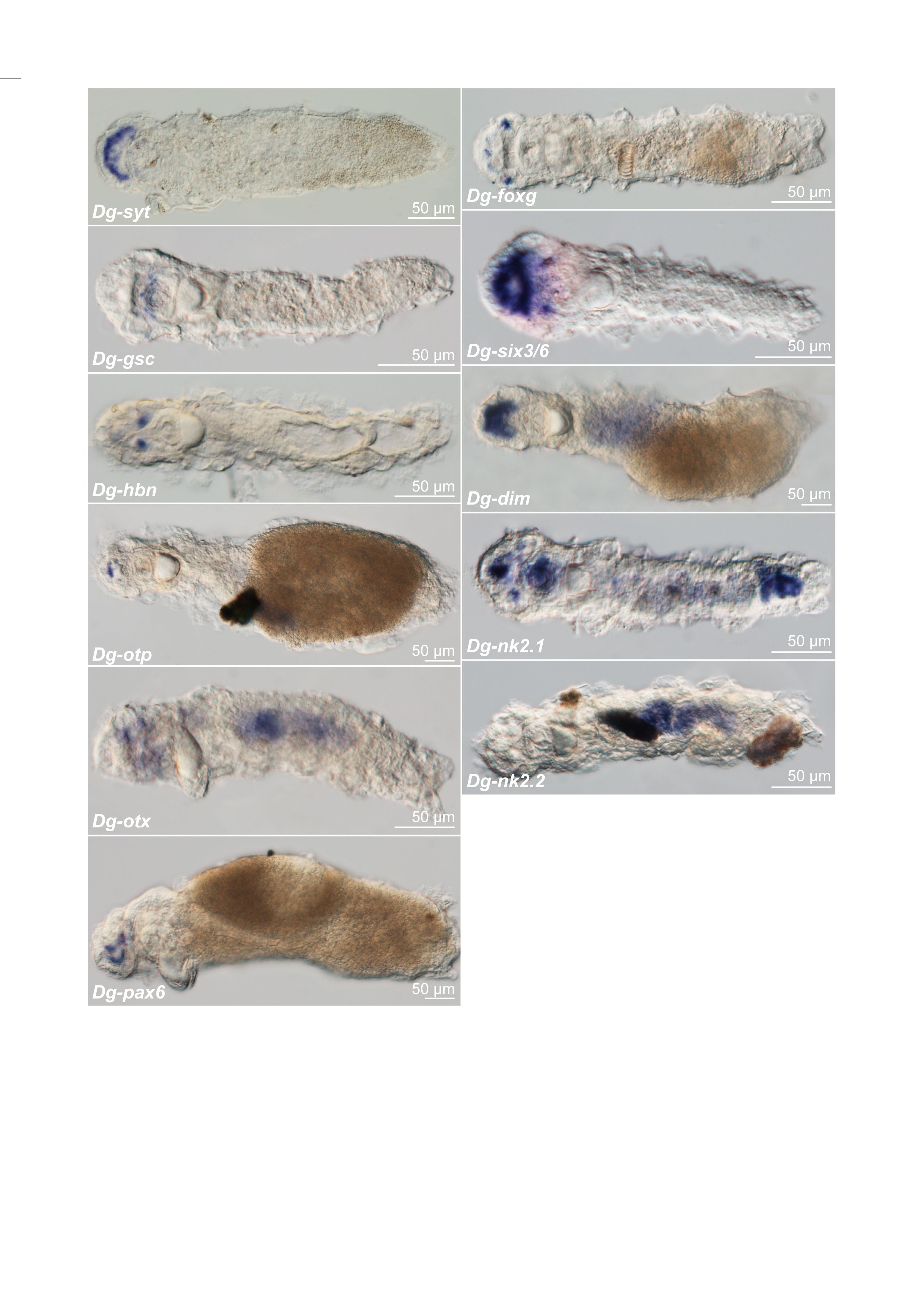

Supplement: Supplementary file 9 — 10.1186/s13227-016-0058-2 Brain-specificity of gene expression patterns for the genes tested. Dg-syt, Dg-gsc, Dg-hbn, Dg-otp, Dg-otx, Dg-pax6, Dg-foxG, Dg-six3/6 and Dg-dim, while Dg-nk2.1 also labels the fore- and hindgut and Dg-nk2.2 is only expressed in the posterior midgut. The light staining in the stomach of specimens labeled with Dg-otx and Dg-dim is an artefact, since precipitation was retained between the stomach content of the animals. Dg-otx and Dg-pax6 are not shown in strict dorsoventral orientation, since the pharyngeal bulb is too extruded to balance the animals dorsoventrally at high magnification. [file 13227_2016_58_MOESM9_ESM.tif]
